# *Arabidopsis* Type II Phosphatidylinositol 4-Kinase PI4Kγ5 Regulates Auxin Biosynthesis and Leaf Margin Development through Interacting with Membrane-Bound Transcription Factor ANAC078

**DOI:** 10.1371/journal.pgen.1006252

**Published:** 2016-08-16

**Authors:** Yong Tang, Chun-Yan Zhao, Shu-Tang Tan, Hong-Wei Xue

**Affiliations:** National Key Laboratory of Plant Molecular Genetics, CAS Center for Excellence in Molecular Plant Sciences, Institute of Plant Physiology and Ecology, Shanghai Institutes for Biological Sciences, Chinese Academy of Sciences, Shanghai, China; Peking University, CHINA

## Abstract

Normal leaf margin development is important for leaf morphogenesis and contributes to diverse leaf shapes in higher plants. We here show the crucial roles of an atypical type II phosphatidylinositol 4-kinase, PI4Kγ5, in *Arabidopsis* leaf margin development. *PI4Kγ5* presents a dynamics expression pattern along with leaf development and a T-DNA mutant lacking *PI4Kγ5*, *pi4kγ5*–1, presents serrated leaves, which is resulted from the accelerated cell division and increased auxin concentration at serration tips. Studies revealed that PI4Kγ5 interacts with and phosphorylates a membrane-bound NAC transcription factor, ANAC078. Previous studies demonstrated that membrane-bound transcription factors regulate gene transcription by undergoing proteolytic process to translocate into nucleus, and ANAC078 undergoes proteolysis by cleaving off the transmembrane region and carboxyl terminal. Western blot analysis indeed showed that ANAC078 deleting of carboxyl terminal is significantly reduced in *pi4kγ5–1*, indicating that PI4Kγ5 is important for the cleavage of ANAC078. This is consistent with the subcellular localization observation showing that fluorescence by GFP-ANAC078 is detected at plasma membrane but not nucleus in *pi4kγ5–1* mutant and that expression of ANAC078 deleting of carboxyl terminal, driven by PI4Kγ5 promoter, could rescue the leaf serration defects of *pi4kγ5–1*. Further analysis showed that ANAC078 suppresses the auxin synthesis by directly binding and regulating the expression of auxin synthesis-related genes. These results indicate that PI4Kγ5 interacts with ANAC078 to negatively regulate auxin synthesis and hence influences cell proliferation and leaf development, providing informative clues for the regulation of *in situ* auxin synthesis and cell division, as well as the cleavage and functional mechanism of membrane-bound transcription factors.

## Author Summary

Transcription factors (TFs) regulate the expression of target genes at nucleus, while interestingly, a few of TFs locate at plasma membrane or ER membrane, stored as resting precursors to secure a rapid response post endogenous or environmental stimuli. Membrane-bound TFs undergo post-translational processing to be liberated and translocate into the nucleus. There are two kinds of liberating mechanisms: regulated intramembrane proteolysis (RIP) by certain membrane-associated proteases and regulated ubiquitin/26S proteasome-dependent processing (RUP). However, the detailed regulatory mechanism is largely unclear. By studying a T-DNA mutant lacking an atypical type II phosphatidylinositol 4-kinase (PI4Kγ5), *pi4kγ5–1*, which presents serrated leaves that resulted from the accelerated cell division and increased auxin concentration at serration tips, we here showed the crucial roles of PI4Kγ5 on the function of membrane-bound TFs, possibly through a phosphorylation-dependent process. Yeast two-hybrid screening identifies ANAC078, a plasma membrane-bound NAC family TF that contains a one-pass transmembrane and undergoes RIP for release, an interacting TF of PI4Kγ5. Biochemical analysis reveal that PI4Kγ5 phosphorylates ANAC078 and further genetic analysis confirmed that PI4Kγ5 is crucial for the cleavage and normal function of ANAC078, indicating that PI4Kγ5-ANAC078 module downregulates auxin level during leaf development (serration). These findings provide new insights into the regulatory and functional mechanism of membrane-bound TFs and *in situ* auxin synthesis and effects during leaf morphogenesis.

## Introduction

Phosphatidylinositol (PI) signaling pathway, as well as the relevant second messenger molecules inositol 1, 4, 5-trisphosphate and various phospholipid molecules, is important for multiple physiological processes in human, animals and plants. PI 4-kinase (PI4K) catalyzes the synthesis of PI 4-phosphate (PI4P) by phosphorylating PI at the 4’ position of the inositol ring [1, 2], and play crucial roles in development and stress responses.

There are twelve PI4K isoforms in *Arabidopsis thaliana*, which can be divided into two subfamilies, type II (PI4Kγ1–8) and type III (PI4Kα1, α2, β1 and β2), according to the structures and molecular weight of proteins (type II PI4Ks are ~70 kDa, smaller than that of type III ones) [3]. Structural and biochemical analysis showed that PI4Kα1 and PI4Kβ1 contain the Pleckstrin Homology (PH) domain that binds to PI4P, and their enzymatic activities are negatively feedback regulated [4]. Physiological studies showed that PI4Kβ1 is involved in the polarized expansion of root hairs by interacting with RabA4b (a Rab GTPase) and *pi4kβ1 pi4kβ2* double mutant presents shorter root hairs compared to wild type [5]. PI4Kβ1 cooperates with PI monophosphate 5-kinase 5 (PIP5K5) to regulate the pectin secretion in tobacco pollen tubes [6]. In addition, PI4Kβ1 is recruited to actin cytoskeleton following binding to PI phosphate kinase 1 [7] and both PI4Kβ1 and PI4Kβ2 act upstream of phospholipase C (PLC) to participate in the cold response [8].

The secondary structure of type II PI4Ks is different from that of type III members. Type II PI4Ks contain PI3/4 kinase domains and variable numbers (none, one, or two) of ubiquitin-like (UBL) domains, while lack the PI-binding domains such as the PH (in PI4Kα) or PPC domains (in PI4Kβ [3]). UBL domain is unique in plants and responsible for protein-protein interaction [9]. According to the numbers of UBL domains and sequence similarity within the kinase domain, type II PI4Ks can be divided into three subgroups: no UBL (PI4Kγ1, γ2, γ8), one UBL (PI4Kγ5, γ6, PI4Kγ7) and two UBLs (PI4Kγ3, PI4Kγ4).

Although type II PI4Ks fail to synthesize PI4P via PI-catalyzed pathway, however, PI4Kγ4 and PI4Kγ7 exhibit protein kinase activity against distinct substrates [9]. PI4Kγ4 directly interacts with and phosphorylates RPN10 (regulatory particle non-ATPase 10) and UFD1 (ubiquitin fusion degradation 1), two proteins of ubiquitin-proteasome system, *in vitro* [9], and studies showed that UBL domain is essential for binding to UFD1 rather than RPN10, suggesting that numbers of UBL domain are related to the function of type II PI4Ks. There are only few reports of the physiological function of type II PI4Ks. *pi4kγ1* mutant presents collapsed mature anthers and most of the pollen grains exhibits irregular shapes [10], while the function of other 7 members needs to be clarified.

The margin development is the final step of leaf morphogenesis, which contributes largely to diverse leaf shapes of higher plants. Leaf margins and leaflet blades are either smooth, serrated (with some indentations, such as *A*. *thaliana*, of which the slightly serrated margin is observed in adult leaves), or lobed (with large outgrowths, such as *A*. *lyrata*), and the position, number and depth of serrations are crucial for leaf margin development. Leaf margin development is regulated mainly by modulating the cell division of leaves [11, 12]. SERRATE (SE), ASYMMETRIC LEAVES1 (AS1), AS2, and KNAT1 (*Arabidopsis* KNOTTED1 LIKE 1) control cell division by regulating the cytokinin concentration of mesophyllic cells [13]; NAC with transmembrane motif 1 (NTM1), Kip-related proteins (KRPs, KRP1/2/3/6), cyclin-dependent kinases (CDKs), and JAGGED regulate margin development by directly regulating cell cycles [14]. In addition, TOUSLED (TSL, a serine/threonine kinase related to chromatin organization) negatively regulates leaf serration [15, 16] and miR319 regulates leaf serration by downregulating the TEOSINTE BRANCHED1, CYCLOIDEA, and PCF (TCP) transcription factors TCP3/4/10/24 [17].

Phytohormone auxin is synthesized in the upper apex and serrations of leaf margin and plays an essential role in leaf margin development, and PIN1-mediated polar auxin transport is required for auxin gradient that initiates serrations [11, 12]. A weak allele of *PIN1* mutant, *pin1-7*, presents smooth leaf margin with no initiation of serrations [12, 18]. In addition, *miRNA164* and its target *CUP-SHAPED COTYLEDON2 (CUC2)* are regulated by auxin and deep serration or smooth margin is observed in *mir164a-4* mutant or *cuc2-3* mutant respectively [19].

Transcription factors (TFs) usually function in the nucleus to regulate the transcription of target genes, however, recent studies showed that some TFs contain transmembrane domains and localize at other subcellular apparatus (such as plasma membrane or ER) [20]. Membrane-bound TFs need to be proteolytically cleaved before translocating into nucleus and studies of how these membrane-bound TFs are cleaved will help to understand the complex regulatory mechanism of gene regulation. We here report the functional characterization of *Arabidopsis* PI4Kγ5 and the molecular mechanisms how PI4Kγ5 regulates leaf margin development through interacting with a membrane-bound NAC (NAM, ATAF1, 2 and CUC2) TF, ANAC078. Our studies showed that PI4Kγ5 is critical for the proteolysis of ANAC078 from membrane, which further regulates the *in situ* auxin synthesis and leaf margin morphogenesis.

## Results

### *PI4Kγ5* is expressed in various tissues and presents a dynamic expression along with rosette leaf development

**Fig 1:**
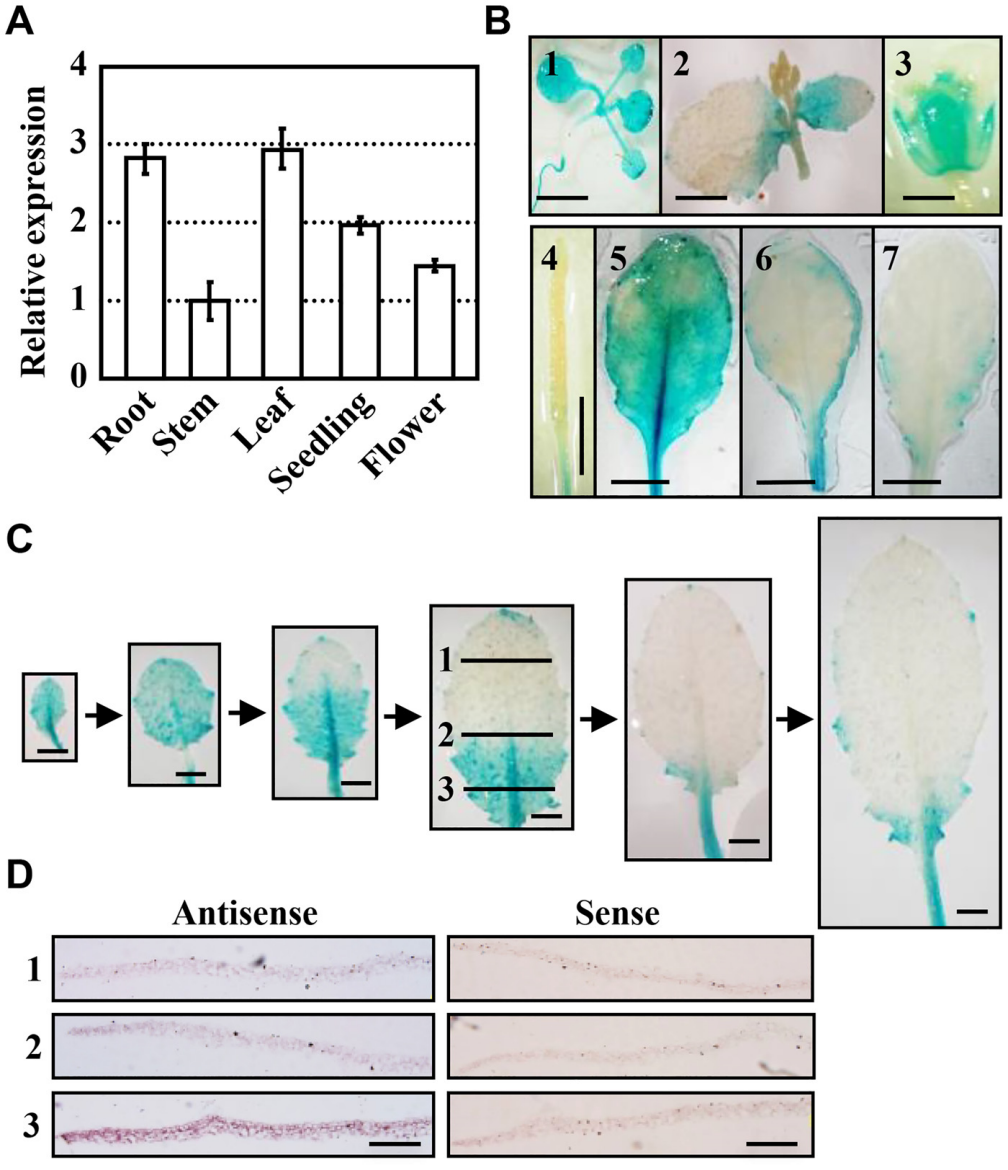
*PI4Kγ5* is expressed in various tissues and exhibits a dynamic expression pattern along with leaf development. A. qPCR analysis revealed the expression of *PI4Kγ5* in various tissues. *ACTIN7* gene was used as an internal reference and transcription level of *PI4Kγ5* in stem was set as 1.0. The experiments were repeated three times and data are presented as means ± SE (n = 3). B. Promoter-GUS fusion analysis showed the *PI4Kγ5* expression in young seedling (1), cauline leaf (2), floral tissue (3), fruit (4), and 5^th^–7^th^ rosette leaves (5–7). Bars = 5 mm. C. Dynamic expression of *PI4Kγ5* along with the 7^th^ rosette leaf development. Representative images were shown. Bars = 2 mm. *D*. *In situ* hybridization analysis of *PI4Kγ5* mRNA in 7^th^ rosette leaf. Different regions including distal (1), central (2) and proximal (3) of leaf (highlighted in C) were sectioned. Bars = 0.5 mm.

Structural analysis showed that *Arabidopsis* type II PI4Kγ5 has one PI3/4-kinase domain and one ubiquitin-like (UBL) domain. Quantitative RT-PCR (qPCR) analysis showed that *PI4Kγ5* is expressed in various tissues, including seedlings, roots, flowers, leaves, and stems (Fig 1A). Further analysis by observing the transgenic lines harbouring β-glucuronidase (GUS) driven by *PI4Kγ5* promoter (*pPI4Kγ5*::*GUS*) confirmed the *PI4Kγ5* expression in seedlings, cauline leaves, cotyledons, sepals and fruits (Fig 1B, 1–4). Detailed observation of the *PI4Kγ5* expression in adult rosette leaves revealed a varied expression of *PI4Kγ5* in different rosette leaves, relatively high in whole blade of the 3^rd^ or 4^th^ leaves, low at the margin of the 5^th^ or 6^th^ leaves, and barely detectable in the serration of the 7^th^ or 8^th^ leaves (Fig 1B, 5–7).

Further observation of the spatial and temporal expression patterns of *PI4Kγ5* along with development of the 7^th^ or 8^th^ leaves, the typical adult leaves, showed that different from that of 3^rd^–6^th^ leaves, *PI4Kγ5* transcript could be detected in the whole blade at early stage (~2 mm), gradually reduced from the top to basal (a size ~ 4 mm—1 cm) and barely detectable at the serration tip when leaves grew from ~1.5–2.5 cm (Fig 1C), which was confirmed by *in situ* RNA hybridization analysis (Fig 1D). The dynamic expression of *PI4Kγ5* along with leaf development suggests a potential role of PI4Kγ5 in leaf morphogenesis.

### *PI4Kγ5* deficiency results in the serrated leaves

**Fig 2:**
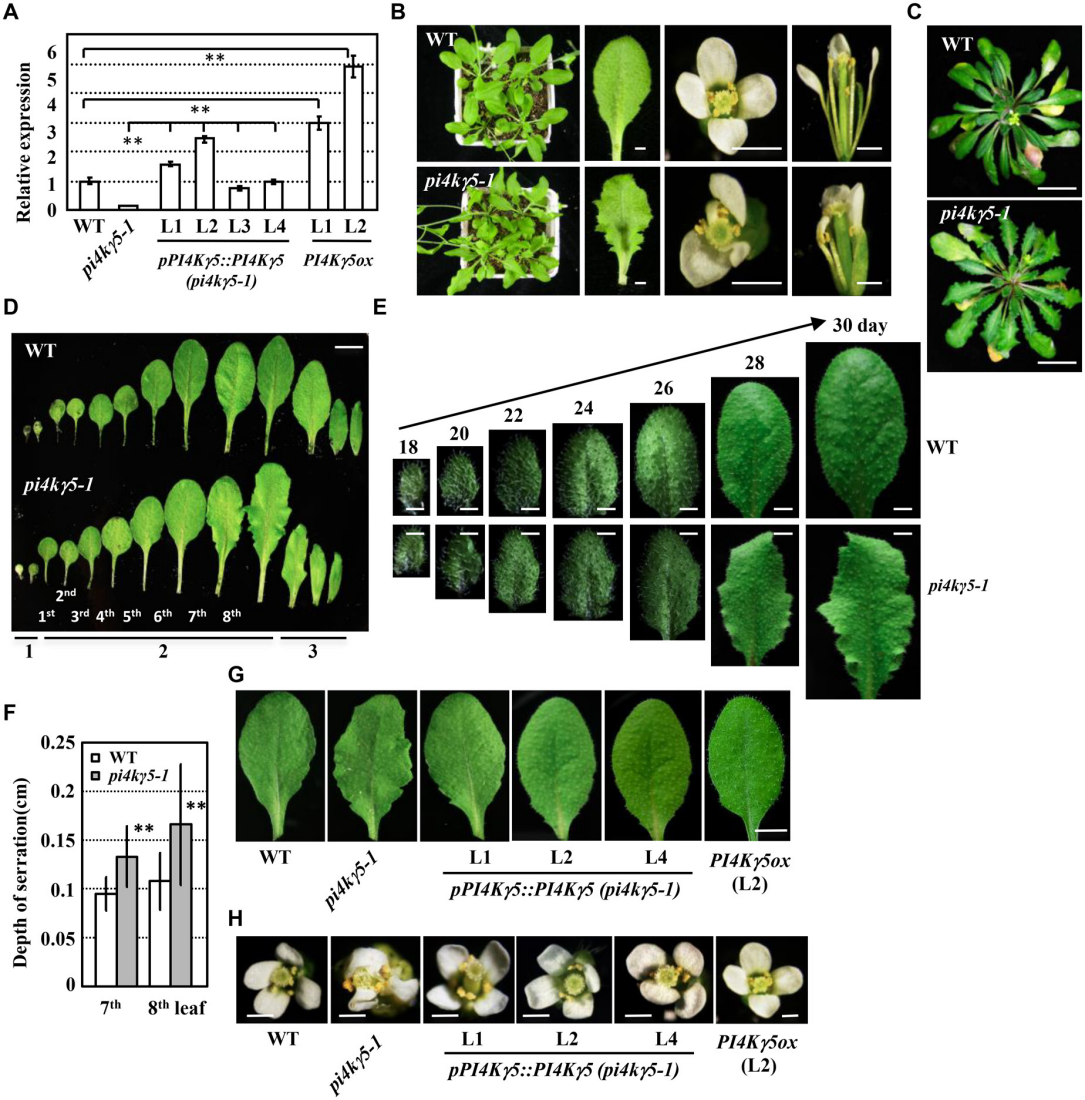
Identification and phenotypic analysis of knockout mutant *pi4kγ5–1*. A. qPCR analysis of the *PI4Kγ5* transcription level in WT and homozygous *pi4kγ5–1* mutant, WT or *pi4kγ5–1* plants expressing *PI4Kγ5* driven by its own native promoter [*pPI4Kγ5*::*PI4Kγ5* (*pi4kγ5–1*) or *PI4Kγ5ox*]. *ACTIN7* gene was used as an internal reference and *PI4Kγ5* expression in WT was set as 1.0. The experiments were repeated three times and data are presented as means ± SE (n = 3). Statistical analysis was performed using a two-tailed Student’s t test (**, p<0.01; *pPI4Kγ5*::*PI4Kγ5* (*pi4kγ5–1*) lines compared to *pi4kγ5–1*; or *PI4Kγ5ox* lines compared to WT). B-C. Phenotypic analysis of 30-day-old (B, under long-day condition) or 60-day-old (C, under short-day condition) *pi4kγ5–1* plants, which showed deep serrated leaves and curved tiny petals. Bars = 5 mm (B) or 2 cm (C). D. *pi4kγ5–1* plants presented deep serrated leaves, especially the 7^th^ and 8^th^ rosette leaves (under long-day condition). The cotyledons (1), rosette leaves (2, the 1^st^ -8^th^ leaves) and cauline leaves (3) were shown. Bars = 1.5 cm. E. Detailed observations showed that 7^th^ and 8^th^ rosette leaves of *pi4kγ5–1* plants exhibit highly serration along with the development and maturation under long-day condition. The 7^th^ leaf was observed at 18, 20, 22, 24, 26, 28, 30 days respectively. Bars = 2 mm. F. Increased serration depth of 7^th^ and 8^th^ leaves of *pi4kγ5–1*. The depth of leaf serrations were measured using 30-day-old plants and statistically analyzed (**, p<0.01). The experiments were repeated three times and data are presented as means ± SD (n = 30). G-H Complementary expression of *PI4Kγ5* in *pi4kγ5–1* recovered the normal leaf shape (G) and petals (H). Representative 7^th^ leaf and flowers of *pi4kγ5–1*, WT, *pPI4Kγ5*::*PI4Kγ5 (pi4kγ5–1)* and *PI4Kγ5ox* plants were shown. Bars = 6 mm (G) or 1 mm (H).

A putative T-DNA insertion mutant, *pi4kγ5–1*, was identified (http://signal.salk.edu/cgi-bin/tdnaexpress [21]). The T-DNA was inserted at the 5’-UTR of PI4Kγ5 (35-bp upstream of ATG, S1A Fig) and qPCR analysis confirmed the deficient *PI4Kγ5* transcription of the homozygous mutants (Fig 2A).

Phenotypic observation showed that *pi4kγ5–1* mutant has more serrated margins in adult leaves under long-day (Fig 2B) or short-day condition (Fig 2C). The serrated margins were mainly observed in the 7^th^ or 8^th^ leaves (Fig 2D), which may result from the differential expression patterns of *PI4Kγ5* in the 7^th^ or 8^th^ leaves compared to that in earlier leaves (Fig 1B). Systemic observation further revealed the altered serration along with 7^th^ or 8^th^ leaf development (Fig 2E), which was confirmed by calculation showing the increased depth of serrations (~20%, Fig 2F) and numbers of serrations (Table 1) in *pi4kγ5–1* leaves. As leaf serration is a key feature of leaf shape, these suggest an important role of *PI4Kγ5* in normal leaf morphogenesis. In addition, two or more petals in one flower were observed being curved, tiny, or even lost under *PI4Kγ5* deficiency (Fig 2B).

**Table 1:** Statistics of serration numbers of the 7^th^ and 8^th^ leaves of wild type and *pi4kγ5–1* plants. Data are presented as means ± SD (n = 30) and statistically analyzed by Student’s t test (**, P< 0.01, compared to those of wild type).

	No. of serrations	
	7^th^ leaf	8^th^ leaf
wild type	2.5±0.7	3.5±1.0
*pi4kγ5–1*	5.9±1.2**	7.3±1.0**

Genetic analysis showed that F2 progeny of heterozygous mutant plants display a 3:1 segregation ratio (normal: serrated, S1 Table), indicating a single T-DNA insertion in the genome. By transforming the *PI4Kγ5* cDNA driven by its own native promoter (*pPI4Kγ5*::*PI4Kγ5*) into *pi4kγ5–1* mutant, the recovered *PI4Kγ5* expression (Fig 2A) resulted in the rescued leaf and floral development (Fig 2G and 2H), confirming the roles of PI4Kγ5 in leaf morphogenesis and margin development. In addition, *Arabidopsis* plants overexpressing *PI4Kγ5* (*PI4Kγ5ox*, driven by CaMV35S promoter, Fig 2A) do not show obvious difference in leaf and flower.

### *PI4Kγ5* deficiency results in the accelerated cell division at leaf margin due to the increased auxin level

**Fig 3:**
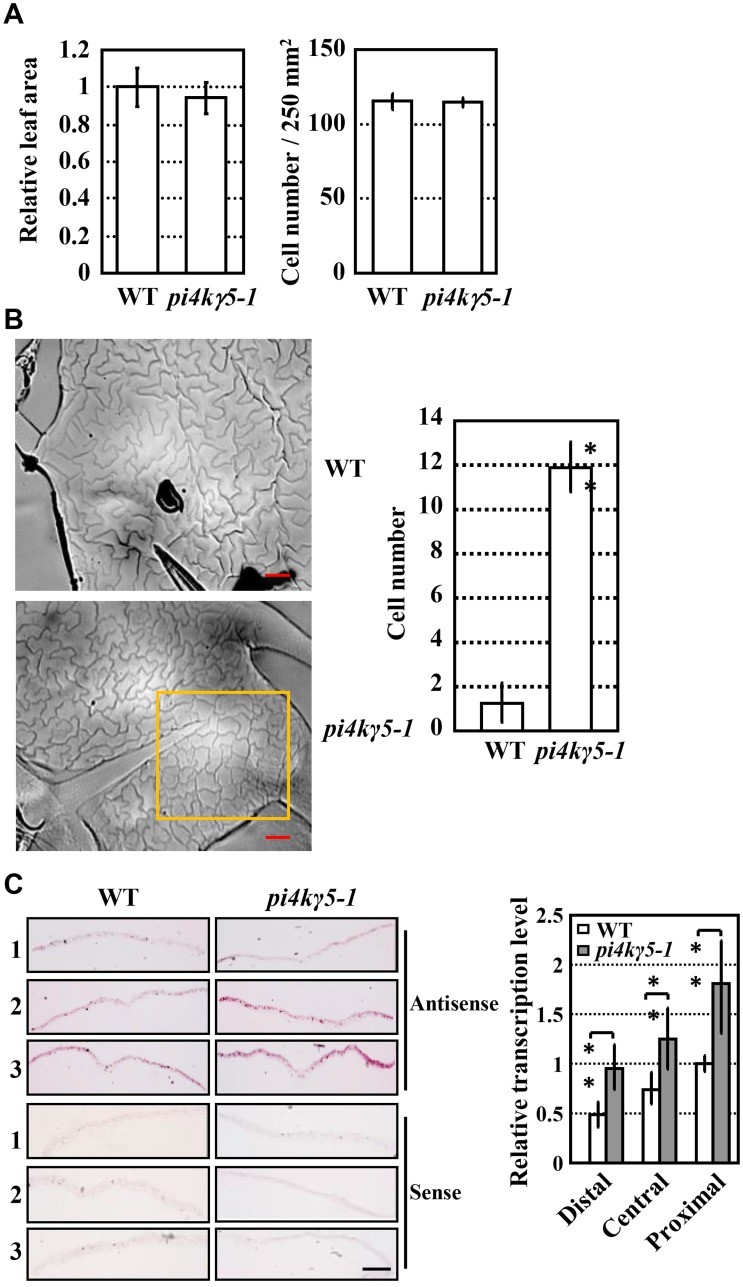
Enhanced cell division at serration tip of *pi4kγ5–1* plants. A. *pi4kγ5–1* plants present similar leaf area (left) and cell numbers (right) compared to those of WT. Palisade cell numbers of same leaf area (250 mm^2^) were measured and statistically analyzed (no significant difference). The experiments were repeated three times and data are presented as means ± SD (n = 20). B. Observation by differential interference contrast microscope (left, bars = 200 μm) and measurement (right) revealed more epidermal cells at premature state (without interdigitated growth) in *pi4kγ5–1* leaves compared to WT (highlighted by squares). The 7^th^ and 8^th^ leaves of 18-day-old plants were measured and statistically analyzed (**, p<0.01). The experiments were repeated three times and data are presented as means ± SD (n = 30). C. *In situ* RNA hybridization analysis (left, bars = 1 mm) and qPCR analysis (right) confirmed the enhanced *H4* mRNA level and hence the cell division at leaf serrations of *pi4kγ5–1*. The 7^th^ and 8^th^ leaves (~6 mm in length) were sectioned as distal (1), central (2) and proximal (3) regions for analysis. The experiments were repeated three times. Data are presented as means ± SD (n = 3) and statistically analyzed (**, p<0.01).

Studies showed that variation of the margin is mostly resulted from the altered cell proliferation in mediolateral direction or along the leaf-lamina contour [22], we therefore investigate whether PI4Kγ5 regulates the cell division at leaf margin. Measurement of the leaf area of the 7^th^ or 8^th^ leaves, and count of palisade cell numbers in the middle of same blade showed that there were no differences between WT and *pi4kγ5–1* (Fig 3A; S1B and S1C Fig), indicating that *PI4Kγ5* deficiency does not lead to the altered cell division in whole adult rosette leaves. However, further observation of the epidermal pavement cells (PCs) at serration revealed obvious difference between WT and *pi4kγ5–1*. In WT plants, epidermal PCs at serration formed a jigsaw-puzzle pattern (few PCs without interdigitated growth at serration tip), while those at the serration tip of *pi4kγ5–1* leaves were much smaller and without interdigitated growth (the slick and sly PCs were considered as just after proliferation, Fig 3B). Histone *H4* gene is highly expressed in actively dividing organ and is used as a criterion to measure the cell division rate [23], and *in situ* hybridization and qPCR analysis of the *H4* gene transcription revealed the enriched *H4* mRNA at leaf margin of basal and middle sections of *pi4kγ5–1* leaves (Fig 3C), which is consistent with the increased number of PCs at serration tip. These results suggested that *PI4Kγ5* deficiency resulted in the enhanced cell division rate at serration tip, which leads to the abnormal leaf margins.

**Fig 4:**
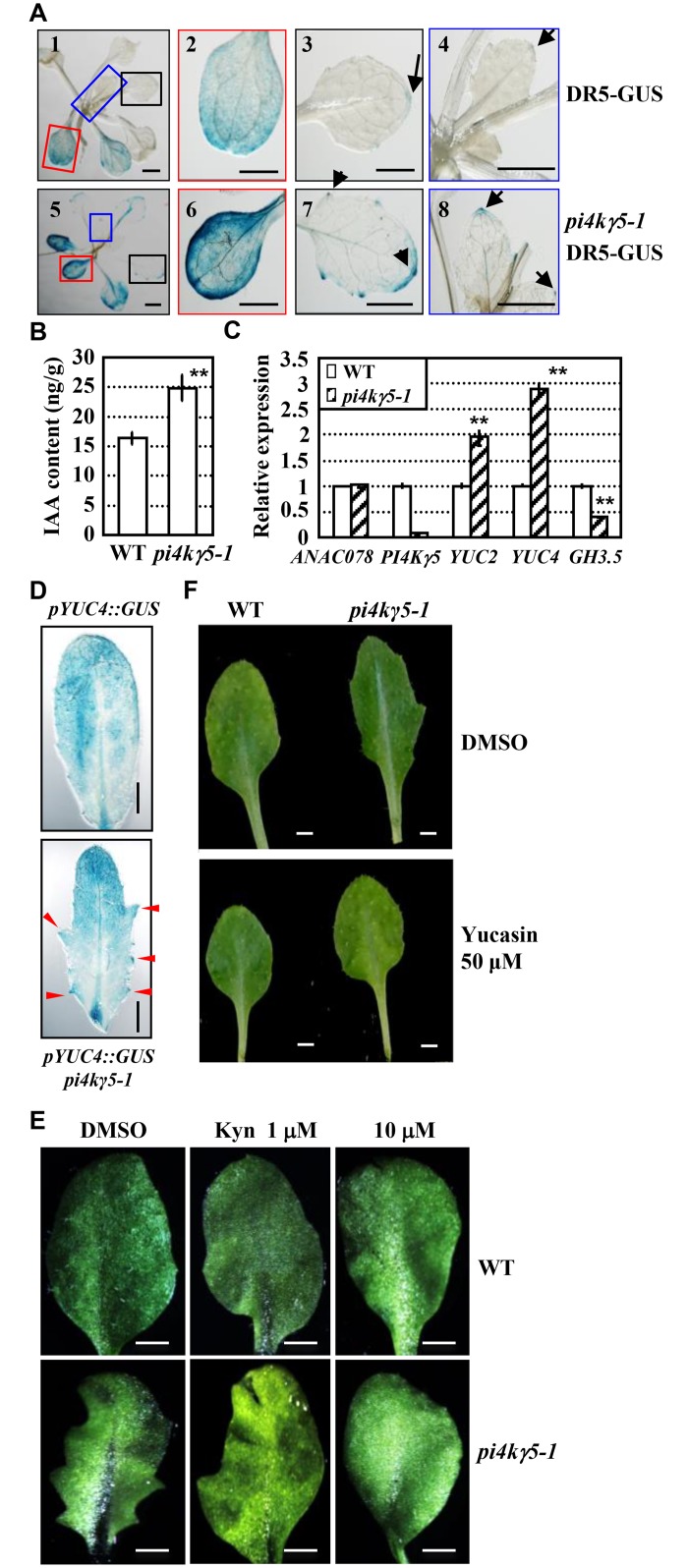
*PI4Kγ5* deficiency results in enhanced auxin biosynthesis. A. Detection of GUS activity in *DR5-GUS* and *pi4kγ5–1 DR5-GUS* lines. Arrows highlight the auxin accumulation at tip of leaf serration and top of leaf. At least 20 lines were analyzed and representative images were shown. Cotyledon (2, 6), 3^rd^ rosette leaf (3, 7), and 5^th^ rosette leaf (4, 8) of whole plant (1, 5) are shown. Bars = 1 mm. B. Quantification analysis by LC/MS revealed the increased IAA amount in the 7^th^ and 8^th^ rosette leaves (~1 cm in length) of *pi4kγ5–1*. Error bars represent SD (n = 3) and statistical analysis was performed by using a two-tailed Student’s t-test (**, p<0.01). C. qPCR analysis of the transcription levels of *YUCs* (*YUC2* and *YUC4*) and *GH3*.*5* in the 7^th^ and 8^th^ rosette leaves (~1 cm in length) of WT and *pi4kγ5–1*. Expression level of the corresponding examined genes in WT was set as 1.0. The experiments were repeated three times and data are presented as means ± SE (n = 3). Statistical analysis was performed by using a two-tailed Student’s t-test (**, p<0.01). D. Analysis of WT or *pi4kγ5–1* seedlings expressing *pYUC4*::*GUS* revealed the much higher *YUC4* expression at *pi4kγ5–1* leaf serrations (highlighted by arrows). The 7^th^ rosette leaf was observed and representative images were shown. Bars = 2 mm. E-F. Treatment by IAA biosynthesis inhibitor L-Kynurenine (L-Kyn, E) or Yucasin (an inhibitor of YUCCA, F) recovered the leaf serration of *pi4kγ5–1*. WT and *pi4kγ5–1* seedlings were grown on MS medium supplemented with L-Kyn (1 or 10 μM) or Yucasin (50 μM) for 30 days and 7^th^ rosette leaf was observed. Representative images were shown. Bars = 1 mm.

Auxin is critical for leaf margin development through regulating cell division and whether auxin accumulation was altered in *pi4kγ5–1* was examined. DR5-GUS, a marker widely used for detection of auxin distribution and accumulation, was transferred into *pi4kγ5–1* through genetic crossing and analysis of the GUS activity revealed the significantly increased auxin content in serration tip and upper apex of *pi4kγ5–1* rosette leaves (Fig 4A; S2 Fig). Measurement by liquid chromatography-mass spectrometry (LC-MS) further confirmed the increased free IAA content in the 7^th^ or 8^th^ *pi4kγ5–1* leaves (Fig 4B). Being consistent, qPCR analysis of the expression of IAA synthesis related genes showed that expressions of *YUCCA2* (*YUC2)* and *YUC4* that encode key enzymes in auxin biosynthesis, are obviously increased, while that of *GH3*.*5* that encodes the auxin-metabolism enzyme, is decreased, in the 7^th^ or 8^th^ *pi4kγ5–1* leaves (Fig 4C). In addition, by using *Arabidopsis* lines harbouring a *pYUC4*::*GUS* reporter [24, 25], higher expression of *YUC4* at the leaf serrations of *pi4kγ5–1* is confirmed (Fig 4D).

Consistent with the increased auxin content at tip of *pi4kγ5–1* rosette leaves, treatment with L-Kynurenine (L-Kyn, a competitive inhibitor of TAA1/TAR to suppress auxin synthesis [25, 26]) and Yucasin (an inhibitor of YUCCA to suppress auxin synthesis [27]) resulted in the reduced auxin content (S3 Fig) and subdued serration at leaf margin (Fig 4E and 4F), further demonstrating that the highly serrated leaf margins in *pi4kγ5–1* was caused by increased auxin content at tip of rosette leaves.

### PI4Kγ5 interacts with and phosphorylates a membrane-bound TF ANAC078

**Fig 5:**
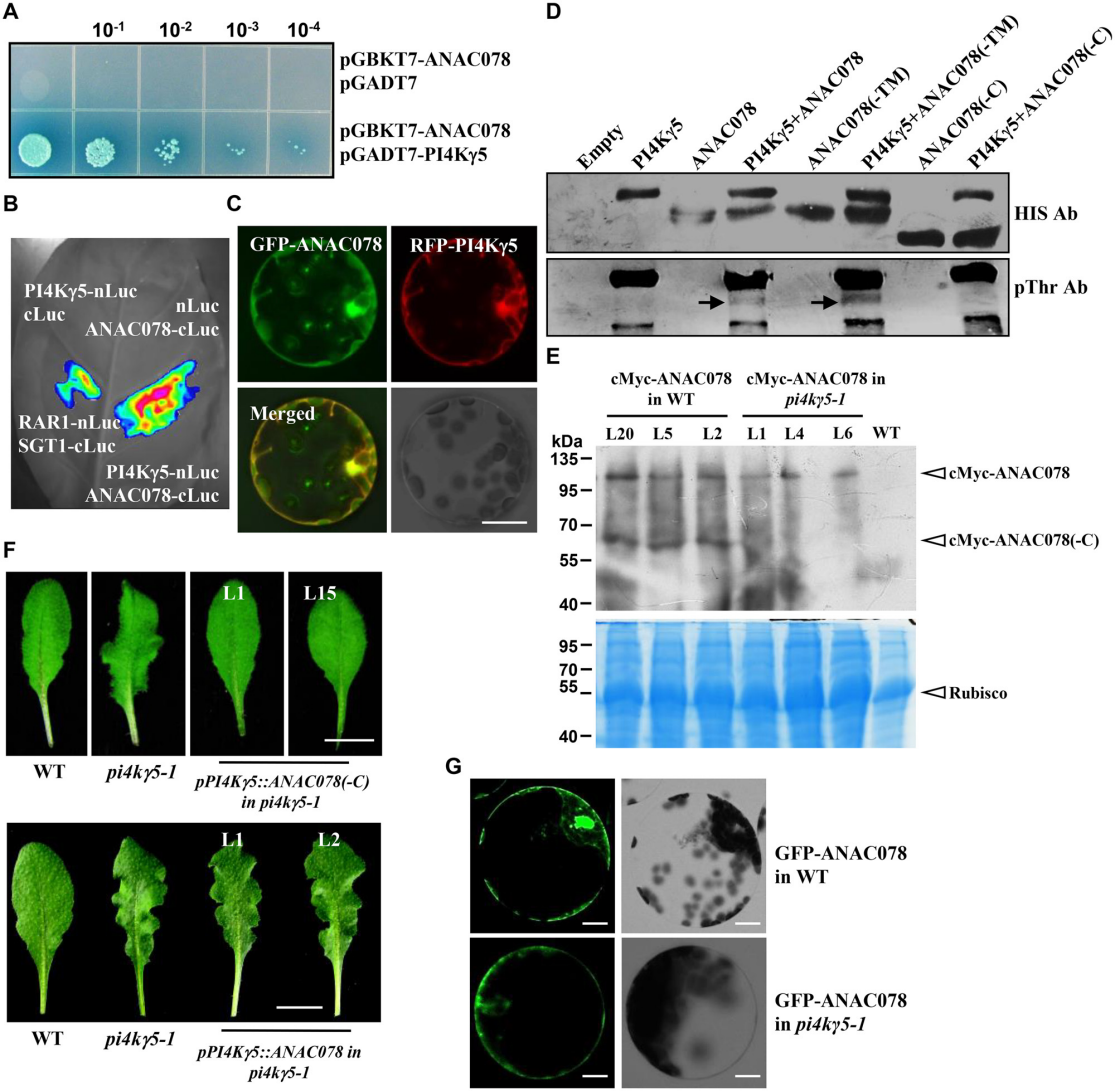
PI4Kγ5 interacts with a membrane-bound TF ANAC078 *in planta* and phosphorylates ANAC078 *in vitro*. A. ANAC078 interacts with PI4Kγ5 in yeast cells. Yeast cells co-transformed with pGBKT7-ANAC078 and pGADT7-PI4Kγ5 were diluted 10, 10^2^, 10^3^ and 10^4^ times and grown on synthetic dropout (SD) medium lacking Leu, Trp, His, and Ade [SD(-Leu-Trp-His-Ade)], supplemented with X-α-gal. Growth of cells was observed after transformation for 2 days and yeast cells co-transformed with pGBKT7-ANAC078 and empty pGADT7 were used as negative control. B. Firefly luciferase complementation assay confirmed the ANAC078-PI4Kγ5 interaction *in planta*. C. Transient expression analysis showed that GFP-ANAC078 co-localizes with RFP-PI4Kγ5 in *Arabidopsis* protoplasts. Bar = 50 μm. D. PI4Kγ5 phosphorylates ANAC078 and ANAC078(-TM) *in vitro*. Recombinantly expressed His-PI4Kγ5, His-ANAC078, His-ANAC078(-C) (deletion of C-terminus), and His-ANAC078(-TM) (deletion of N-terminal transmembrane region) were used for kinase assay. Anti-His and anti-pThr antibodies were used to detect the protein input (upper panel) and phosphorylated proteins (bottom panel), respectively. E. Western blot analysis revealed that ANAC078(-C) amount was significantly reduced in *pi4kγ5–1*. The 7^th^ and 8^th^ rosette leaves (~100 mg) of WT or *pi4kγ5–1* plants expressing *cMyc-ANAC078* (*cMyc-ANAC078* in WT; or *cMyc-ANAC078* in *pi4kγ5–1*) were used for protein extraction and western blot analysis (upper panel). A mouse cMyc antibody was used to detect the cMyc-ANAC078 or cMyc-ANAC078(-C) proteins. CBB staining indicated the equal protein loading (bottom panel). F. Expression of *ANAC078(-C)* driven by *PI4Kγ5* promoter rescued the leaf serration of *pi4kγ5–1*, while full *ANAC078* could not. The 7^th^ rosette leaf was shown. Bars = 1 cm. G. Fluorescence observation revealed the fluorescence of GFP-ANAC078 in the nucleus and plasma membrane of WT protoplasts, while only in plasma membrane but not nucleus of *pi4kγ5–1* protoplasts. Serration of the 7^th^ and 8^th^ rosette leaves of 28-day-old WT or *pi4kγ5–1* plants were used for protoplast preparation. Bar = 20 μm.

To study the mechanism how PI4Kγ5 regulates the *in situ* auxin biosynthesis and hence the leaf margin development, yeast two-hybrid screening was performed using whole PI4Kγ5 protein as bait to identify the interacting proteins/candidate substrates of PI4Kγ5. Of the obtained positive clones, two of them (S4A Fig) encode fragments of ANAC078 (271–410 aa), a membrane-bound transcription factor. The PI4Kγ5-ANAC078 interaction was confirmed by examining the α-Gal activity (Fig 5A) and further firefly luciferase complementation assay *in planta* (Fig 5B). In addition, transient expression of RFP-PI4Kγ5 and GFP-ANAC078 fusion proteins in *Arabidopsis* protoplasts showed that RFP-PI4Kγ5 and GFP-ANAC078 partially co-localize with each other at cell membrane and nucleus (Fig 5C).

Considering that three type II PI4K members (PI4Kγ1, 4, 7) are serine/threonine protein kinases [9], it is supposed that PI4Kγ5 might function as a protein kinase to phosphorylate and regulate the target proteins. We then investigated whether PI4Kγ5 could phosphorylate ANAC078. *In vitro* phosphorylation assay using anti-phospho-Threonine (pThr) antibody revealed that PI4Kγ5 exhibits protein kinase activity and directly phosphorylates ANAC078 (Fig 5D).

### PI4Kγ5 is crucial for ANAC078 functions

Previous studies showed that membrane-bound NAC precursors undergo proteolytic cleavage of TM and C-terminus to translocate into nucleus and ANAC078 might form three distinct polypeptides: the full membrane-bound form (1–567 aa), the nuclear form deletion of transmembrane (TM) region (ANAC078-TM, 1–539 aa) and the nuclear form deletion of C-terminus (ANAC078-C, 1–399 aa) [28–31] (S4B Fig). Further *in vitro* phosphorylation assay showed that PI4Kγ5 could phosphorylate ANAC078 and ANAC078(-TM), but not ANAC078(-C) (Fig 5D), which confirmed ANAC078 a substrate of PI4Kγ5 and phosphorylation of ANAC078 occurs at the C-terminus and suggested that PI4Kγ5 may involve in the regulation of ANAC078 cleavage and translocation to nucleus.

Indeed, by transforming cMyc-ANAC078 fusion protein into WT or *pi4kγ5–1*, western blot assay showed that compared to WT, much less ANAC078(-C) is detected in *pi4kγ5–1* (Fig 5E), suggesting the proteolytic process of cMyc-ANAC078 is largely suppressed. Interestingly, expression of *ANAC078(-C)* driven by *PI4Kγ5* promoter [*pPI4Kγ5*::*ANAC078(-C)*] in *pi4kγ5–1* could rescue the highly serrated *pi4kγ5–1* leaf, while expression of full *ANAC078* driven by *PI4Kγ5* promoter (*pPI4Kγ5*::*ANAC078*) not (Fig 5F), confirming the important role of PI4Kγ5 on normal function of ANAC078.

Being consistent, additional observation of the localization of GFP-ANAC078 in WT or *pi4kγ5–1* mutant showed that the fluorescence was detected in plasma membrane and nucleus in WT serration, while the fluorescence was observed in plasma membrane only in serration of *pi4kγ5–1* mutant (Fig 5G), indicating the presence of full membrane-bound form of ANAC078 in *pi4kγ5–1* and further demonstrating that PI4Kγ5 is required for ANAC078 cleavage and proteolytic activation.

Previous studies showed that ANAC078 regulates flavonoid biosynthesis under high-light [30] and our preliminary analysis revealed that similar to *anac078* mutant, genes related to flavonoid biosynthesis or transcription factors regulating the expression of these genes presented suppressed expression in *pi4kγ5–1* plants under high-light (S5 Fig), suggesting that PI4Kγ5 may involve in flavonoid biosynthesis through regulating ANAC078.

qPCR analysis showed that *ANAC078* is highly expressed in leaves and stems (S6A Fig). Genetic analysis showed that *anac078 pi4kγ5–1* double mutant (S6B and S6C Fig) presented phenotype similar as *pi4kγ5–1* (S6D Fig), demonstrating the genetic epistasis of *PI4Kγ5*. By the way, *anac078* mutant presents indistinguishable growth from WT and plants overexpressing ANAC078 has smaller rosette leaves, but no serrated margins in adult leaves (S6E Fig).

### ANAC078 directly regulates auxin biosynthesis and cell division

**Fig 6:**
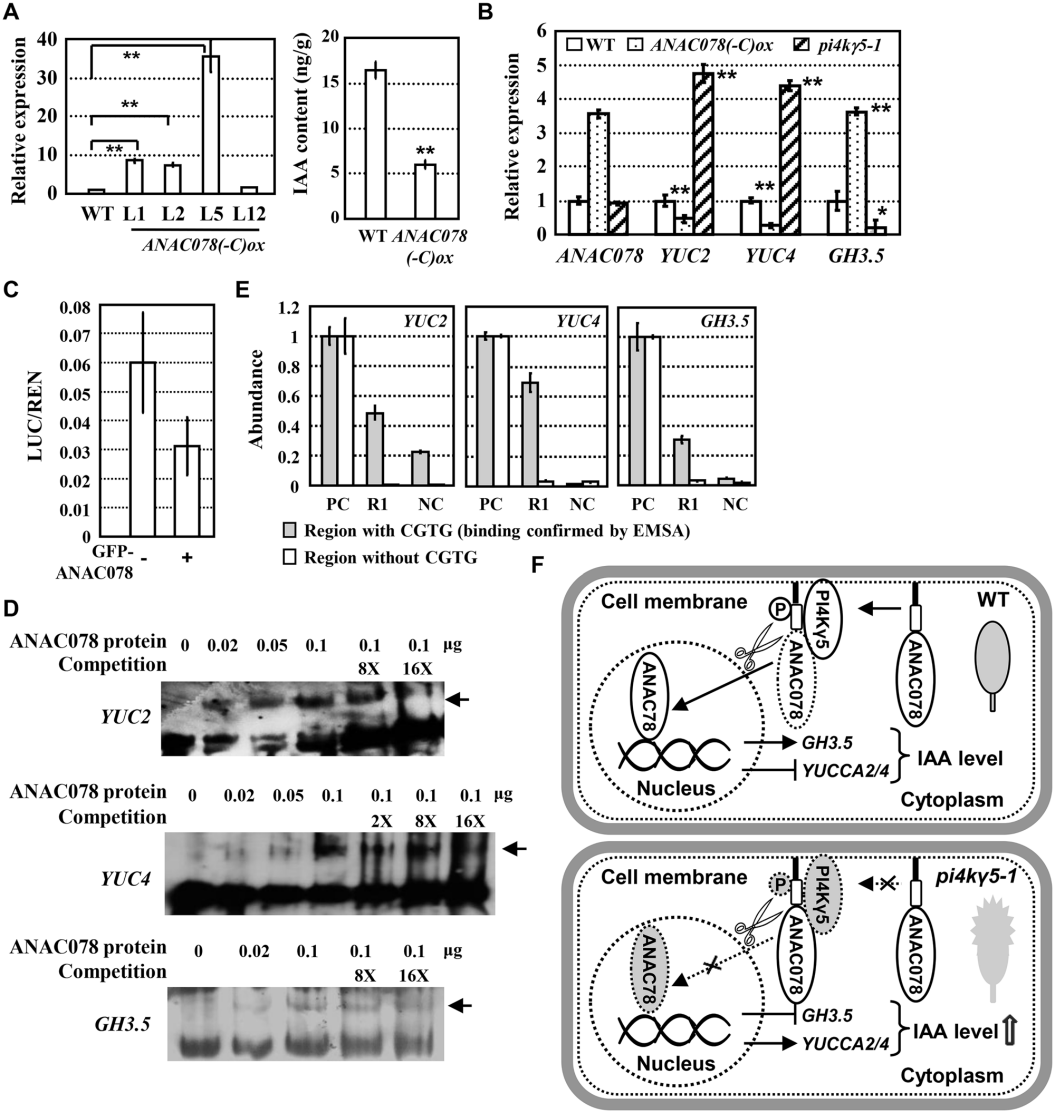
ANAC078 negatively regulates auxin synthesis by directly binding to the promoters of *YUC2*, *YUC4* and *GH3*.*5*. A. qPCR analysis confirmed the expression of *ANAC078(-C)* in WT [*ANAC078(-C)ox*] (left), which results in the decreased IAA amount (right). *ACTIN7* gene was used as an internal reference and *ANAC078* transcription level in WT was set as 1.0. IAA content was measured by LC/MS using the 7^th^ and 8^th^ rosette leaves (~1 cm in length) of 20-day-old plants. The experiments were repeated three times and statistically analyzed (**, p<0.01). Data are presented as means ± SD (n = 3). B. qPCR analysis revealed the opposite expressions of auxin-synthesis related genes (*YUC2*, *YUC4*, and *GH3*.*5*) in *ANAC078(-C)ox* and *pi4kγ5–1* plants, indicating the negative effects of ANAC078(-C) in auxin synthesis. *ACTIN7* gene was used as an internal reference and transcription level of tested gene in WT was set as 1.0. The experiments were repeated three times and statistically analyzed (**, p<0.01). Data are presented as means ± SE (n = 3). C. ANAC078 presents transcriptional repression activity. Transient transcription dual luciferase (Dual-LUC) assay was performed by using *YUC2* promoter in the absence or presence of GFP-ANAC078. Relative LUC activities normalized to the REN activity (LUC/REN) was calculated. The experiments were repeated three times and data are presented as means ± SD (n = 3). D. EMSA assays showed that ANAC078 directly binds to the promoters of *YUC2*, *YUC4*, and *GH3*.*5*. Arrows indicated the shifted bands of DNA fragments. E. ChIP assay confirmed that ANAC078 directly binds to the promoters of *YUC2*, *YUC4*, and *GH3*.*5*. qPCR was performed to detect the DNA abundance. Input was added as positive control (PC) and IP samples without antibody were used as negative control (NC). R1, IP samples by using GFP antibody. The amplified DNA abundance using PC as template was set as 1.0. The experiments were repeated three times and data are presented as means ± SE (n = 3). F. A hypothetic model how PI4Kγ5-ANAC078 module regulates auxin synthesis and leaf development. ANAC078 is an important negative regulator of auxin synthesis by down-regulating auxin-synthesis genes *YUC2* and *YUC4*, or up-regulating auxin-metabolism gene, *GH3*.*5*. Proteolysis of transmembrane region and C-terminal of ANAC078 results in the translocation of ANAC078(-C) into nucleus, while interaction with PI4Kγ5 is crucial for ANAC078 proteolysis, possibly through phosphorylation, to maintain the normal ANAC078 cleavage and proper auxin levels in rosette leaves, leading to the fine-controlled leaf margin development and morphogenesis (upper panel). In *pi4kγ5–1* mutant, defective interaction with PI4Kγ5 results in the suppressed ANAC078 cleavage, possible due to the reduced phosphorylation, and hence enhanced auxin synthesis, which leads to the promoted cell proliferation at leaf teeth and highly serrated rosette leaves (bottom panel). This provides a complex regulation of *in situ* auxin synthesis and leaf development.

As expressions of *YUC2* and *YUC4* are increased in *pi4kγ5–1*, it is hypothesized that ANAC078 might negatively regulate the auxin synthesis and cell division. Indeed, measurement by LC/MS showed the reduced auxin content (~60% reduction) in rosette leaves of plants overexpressing *ANAC078(-C)* (Fig 6A). Further qPCR analysis confirmed the suppressed expressions of *YUC2* and *YUC4* and increased expression of *GH3*.*5* in the 7^th^ and 8^th^ *ANAC078(-C)ox* leaves (Fig 6B). The opposite auxin content and expressions of auxin synthesis related genes further indicated the negative regulation of ANAC078 function by PI4Kγ5.

Previous studies showed the transcriptional activation activity of ANAC078 [31]. Considering that ANAC078 overexpression results in the increased or decreased expression of downstream genes, a dual luciferase assay using *YUC2* promoter further confirmed the transcriptional repression activity of ANAC078 (Fig 6C). In addition, NAC transcription factors might regulate the expression of target genes by recognizing a core cis-element CGTG in the promoter regions [30–32], and predication analysis revealed the presence of one or two CGTG elements in the promoter regions of *YUC2*, *YUC4* and *GH3*.*5*. Electrophoretic mobility shift assay (EMSA) was then performed and results demonstrated that ANAC078 could directly bind the promoter fragments of *YUC2*, *YUC4* and *GH3*.*5* (Fig 6D). Further chromatin immunoprecipitation (ChIP) assay by using the transgenic plants expressing YFP-ANAC078 fusion protein revealed the significant enrichment of the DNA fragments of *YUC2*, *YUC4* and *GH3*.*5* promoter regions containing CGTG elements (Fig 6E), confirming *YUC2*, *YUC4* and *GH3*.*5* as the direct targets of ANAC078 *in planta*.

## Discussion

### The PI4Kγ5-ANAC078 module is essential for ANAC078 function in regulating *in situ* auxin synthesis and cell division

Our studies identified a regulatory pair of factors that are crucial in regulating auxin biosynthesis and cell division during leaf margin development. In addition to the feedback transcriptional network by miR164-CUC2 and PIN1-mediated auxin transport [12, 33, 34], our results further demonstrated that ANAC078 and PI4Kγ5 synergistically regulate the *in situ* auxin biosynthesis and cell division.

The leaf margin morphogenesis is initiated by serration pattern determination and formed with differential cell proliferation at the mediolateral direction or along the leaf-lamina contour [22]. Transcription of *PI4Kγ5* is relatively high at early stage and decreased along with the leaf development, and is finally restricted at leaf margin, especially the serration tips of matured leaves, which is consistent with the PI4Kγ5 effect in regulating cell division at leaf margin by regulating auxin synthesis. It is hypothesized that PI4Kγ5 interacts with membrane-bound ANAC078 to promote its proteolytic processing, possibly through phosphorylation, to maintain the normal auxin concentration, and hence regulates the final leaf shape with weak serrations. Deficiency of PI4Kγ5 results in the defective interaction and suppressed ANAC078 cleavage, resulting in the enhanced auxin synthesis and promoted cell proliferation at leaf teeth and exhibiting highly deep serrations (Fig 6F). These present a novel mechanism how *in situ* auxin synthesis and leaf serration/leaf morphogenesis are regulated, in addition to the PIN1-mediated polar transport [18].

*pi4kγ5–1* mutant has highly serrated 7^th^ and 8^th^ rosette leaves, while other rosette leaves (1^st^–6^th^) are similar to WT under long day condition (expression pattern of *PI4Kγ5* in 7^th^ and 8^th^ rosette leaves is different from which in other leaves as well), indicating that PI4Kγ5-ANAC078 functions dependently on the plant intrinsic timing cues, such as the microRNA156-SPLs pathway [11]. In addition, expression of *ANAC078(-C)* driven by *PI4Kγ5* promoter rescues the *pi4kγ5–1* phenotype, but driven by CaMV35S promoter not, suggesting that the expression pattern of *PI4Kγ5* is essential for its function.

### PI4Kγ5 is crucial for ANAC078 function

Membrane-bound transcription factors undergo post-translational processing to be released and activated, presenting a rapid responsive mechanism to regulate the gene expression. Regarding the two kinds of liberating mechanisms: regulated intramembrane proteolysis (RIP) by certain membrane-associated proteases and regulated ubiquitin/26S proteasome-dependent processing (RUP) [20], the mammalian sterol response element binding proteins (SREBPs) are typical examples for RIP. SREBPs have two transmembrane spans and are tethered at ER membrane, forming a complex with SREBP cleavage activation protein (SCAP, a sterol sensor), which could shuttle SREBPs from ER to Golgi under stimuli for further proteolytic release by proteases [20, 35].

Recent studies showed that the ER tethered ETHYLENE INSENSITIVE2 (EIN2) underwent phosphorylation and proteolysis to translocate into nucleus [36]. NAC transcription factor NTM1 was post-translationally modified by RIP to enter into nucleus [28] and ANAC078 undergoes a similar post-translational proteolytic cleavage. However, the detailed regulatory mechanism, especially the regulating components during this process, is still unknown. Our studies reveal that PI4Kγ5 is crucial for ANAC078 proteolysis, presenting a novel regulatory mechanism for membrane-bound transcription factors. In addition, whether PI4Kγ5 involves in a general RIP process and detailed machinery for this translocation should be addressed in the future.

Our studies showed that PI4Kγ5 functions as a protein kinase rather than a PI kinase, presenting a new clade of the PIKK (phosphoinositide kinase related kinase) family. In animals, PIKK members, ATM (ataxia-telangiectasia mutated) and ATR (ATM- and Rad3-related), function as protein kinases that are essential for DNA double-strand break (DSB) repair [37]. Plant type II PI4K members contains plant-specific UBL domain, via which PI4Kγ4 interacts with RPN10 and UFD1 that are related to 26S proteasome [9]. PI4Kγ5 functions as a protein kinase and interacts with ANAC078 to regulate its cleavage, providing additional evidence how type II PI4Ks confer their functions in plants.

PI4Kγ5 is important for the normal function of ANAC078 and PI4Kγ5 is required for ANAC078 cleavage and proteolytic activation, however, as the phosphorylation sites of ANAC078 aren’t known yet, we still can’t exclude other possible regulations of ANAC078, although it seems very likely through phosphorylation mediated by PI4Kγ5.

### *In situ* auxin biosynthesis is important for leaf morphogenesis

Although many studies indicated that auxin plays important roles in leaf morphogenesis, still little is known how local auxin regulates leaf serration or leaf margin development. Previous studies showed that polar auxin transport is important for maintaining the auxin levels at leaf serrations [18] and auxin is mainly distributed at leaf tips and absent from the leaf margin before serration outgrowth, and then emerges at serration tips when serration initiates [12]. Deficiency of auxin efflux carrier, PIN1, results in the loss of local auxin activity maxima and smooth leaves without serrations [12, 18], indicating the importance of auxin gradient in leaf serration. In addition, the YUCCA enzymes are essential for leaf development [38], however, whether they function at leaf serrations is rarely known. Our studies indicated that ANAC078 directly regulates *in situ* auxin biosynthesis through binding to the promoters of *YUCCA*s and *GH3*.*5*, which is crucial for suppressing exaggerated leaf serrations, suggesting that both polar transport and *in situ* auxin synthesis contribute to local auxin activity maxima and serrations outgrowth. *ANAC078* is expressed in rosette leaves and much lower expression of *PI4Kγ5* in the serration of the 7^th^ or 8^th^ leaves are consistent with the expressions of *YUC2/YUC4* and hence auxin accumulation in leaves. Interestingly, the spatial and temporary expression pattern of *PI4Kγ5* dominates where ANAC078 functions and hence regulates auxin synthesis. This inhibitory pathway for *in situ* auxin synthesis may tune with the polar transport to control the leaf serration and leaf shape.

## Materials and Methods

### Plant materials and growth conditions

All *Arabidopsis thaliana* plants were in Columbia-0 (Col-0) background. The T-DNA insertion mutants, *pi4kγ5–1* (SALK_026136) and *anac078-1* (SALK_025098) were obtained from the *Arabidopsis* Biological Resource Center (ABRC). The *Arabidopsis* line harbouring *pYUC4*::*GUS* [21] was kindly provided by Prof. Yunde Zhao at UCSD.

Seeds were surface-sterilized and sown on plates containing Murashige and Skoog (MS) medium (Duchefe Biochemie, The Netherlands). After stratification at 4°C for 4 days, seedlings were grown in phytotron with a 16-h light/ 8-h dark cycle (22°C) for normal growth and seed harvesting.

### Identification of T-DNA mutants

Mutant *pi4kγ5–1* carrying a T-DNA insertion at the 5’-UTR and was confirmed by PCR amplification using primers PI4Kγ5-1/PI4Kγ5–2. Transcription level of *PI4Kγ5* was examined by qPCR using primers PI4Kγ5-3/PI4Kγ5–4. T-DNA insertion of mutant *anac078-1* was confirmed by PCR amplification using primers ANAC078-1/ANAC078-2. Primers ANAC078-3/ANAC078-4 were used to exam the transcription level of *ANAC078*. All primers used in this study were listed in S2 Table and all constructs were confirmed by sequencing.

### Promoter-reporter gene fusion studies

Promoter of *PI4Kγ5* (-2300 bp upstream of ATG) was amplified using genomic DNA as template (primers PI4Kγ5-5/PI4Kγ5–6) and subcloned into a modified pCAMBIA1300 vector with a GUS reporter [39]. Resultant construct was transformed into *Arabidopsis* by floral dip methods [40] and histochemical assay of GUS activities was performed according to previous description [41]. Samples were observed using DIC microscopy (Nikon SMZ1500) and representative images were shown.

### Constructs and plant transformation

Full-length cDNAs of *PI4Kγ5* (RAFL04-13-O14) and *ANAC078* (RAFL09-67-D01) were obtained from RIKEN BRC. For expression of *ANAC078(-C)* in WT or *pi4kγ5–1*, coding sequences of *ANAC078(-C)* (1–1197 bp) were amplified (primers ANAC078-19/ANAC078-20) and subcloned into the pCambia1301p vector.

For expression of *ANAC078(-C)* driven by *PI4Kγ5* promoter, coding sequences of *ANAC078(-C)* were firstly amplified by PCR (primers ANAC078-19/ANAC078-20) and subcloned into a modified pCambia1300 vector [36], then the *PI4Kγ5* promoter was amplified by PCR (primers PI4Kγ5-5/PI4Kγ5–6) and subcloned into the resultant plasmid.

For expression of *cMyc-ANAC078* in WT or *pi4kγ5–1*, coding sequence of *ANAC078* was amplified (primers ANAC078-7/ANAC078-8) and subcloned into a modified pEGAD-4XcMyc vector with N-terminal fusion.

For expression of *GFP-ANAC078* in WT or *pi4kγ5–1*, coding regions of *ANAC078* (primers ANAC078-5 and ANAC078-6) was amplified and subcloned into vector pA7 (N-terminus fusion with GFP), resulting in the GFP-ANAC078 fusion constructs. Resultant construct was transiently expressed in *Arabidopsis* protoplasts by PEG/CaCl_2_ methods [46]. Fluorescence was observed by confocal laser scanning microscopy (Olympus FV1000).

Plant transformation was performed by floral dip methods.

### Quantitative real-time RT-PCR (qPCR) analysis

qPCR analysis was performed to examine the *PI4Kγ5* transcription in various tissues, expression of *YUC2*, *YUC4* and *GH3*.*5* in different mutants or transgenic lines, expressions of *PI4Kγ5*, *ANAC078*, *ANAC078(-C*), and *ANAC078(-TM)* in transgenic plants (primers YUC2-1/YUC2-2, YUC4-1/YUC4-2, GH3.5-1/GH3.5–2, PI4Kγ5-3/PI4Kγ5–4, ANAC078-3/ANAC078-4), expressions of *H4* in *pi4kγ5–1* and WT (primers H4-3/H4-4), and expressions of *PAP1*, *TT2*, *TT8*, *CHI*, *CHS* and *F3H* in *pi4kγ5–1*, *anac078*, and WT (primers PAP1-1/PAP1-2, TT2-1/TT2-2, TT8-1/TT8-2, CHI-1/CHI-2, CHS-1/CHS-2, F3H-1/F3H-2 [30]). Total RNAs were extracted and used to synthesize the cDNAs by reverse transcription. The *ACTIN7* (*AT5G09810*) was amplified (primers ACTIN7-1/ACTIN7-2) and used as an internal reference. Primers are listed in S2 Table.

### Free IAA contents measurement by liquid chromatography/ mass spectrometry (LC/MS)

500 mg of 7^th^ and 8^th^ rosette leaves were frozen in liquid nitrogen and ground to a fine powder for free IAA content measurement by Thermo TSQ Quantum Ultra LC-MS-MS system [42].

### *In situ* hybridization analysis

Gene-specific coding regions of *PI4Kγ5* and *H4* were amplified by PCR (primers PI4Kγ5-3/PI4Kγ5–4, or H4-1/H4-2) and subcloned into pGEM-T easy vector (Promega, USA). The sense and antisense probes were transcribed *in vitro* under T7 promoter with RNA polymerase using a DIG RNA labeling kit. The 7^th^ rosette leaf of WT or *pi4kγ5–1* at different developmental stages were fixed in a formaldehyde solution (4%), dehydrated through an ethanol series, embedded in paraffin (Sigma-Aldrich, USA), and sectioned at 10 mm. *In situ* hybridization was performed according to previous description [43].

### Recombinant expression of PI4Kγ5 and *in vitro* kinase assay

Coding regions of *PI4Kγ5* (primers PI4Kγ5-9/PI4Kγ5–10) and *ANAC078(-C)* (primers ANAC078-7/ANAC078-14) were amplified and subcloned into vector pET32a (Novagen, Germany) respectively. Similarly, coding regions of *ANAC078* (primers ANAC078-7/ANAC078-8) and *ANAC078(-TM)* (primers ANAC078-7/ANAC078-13) were amplified and subcloned into pET28a vector respectively.

Proteins were recombinantly expressed in *E*. *coli* (strain *BL21*) by supplement with 1 mM IPTG (Isopropyl β-D-1-Thiogalactopyranoside, 16°C, 12 h) and then purified using Ni-NTA His binding resin (Novagen, Germany) according to the manufacturer’s protocols. Kinase activity assay was carried out according to previous description [9] with few modifications. Assay was initiated by adding 6 μl of kinase solution containing recombinant His-PI4Kγ5 protein (1 μg) in a total volume of 40 μl containing 50 mM Tris-HCl (pH 7.5), 10 mM MgCl_2_, 1 mM EGTA, 10 mM ATP, and 10 μg of substrate [recombinant His-ANAC078, His-ANAC078(-C), or His-ANAC078(-TM)]. Reactions were incubated at 37°C for 3 h and terminated by adding 2X SDS loading buffer. After boiled for 5 min, the products were fractionated by SDS-PAGE and transferred to a PVDF membrane (PerkinElmer, USA) by semi-dry blotting. After washing with TBST buffer (0.1 M Tris-HCl, 0.15 M NaCl, 0.05% Tween-20, pH 7.5) for three times, the phosphorylation of substrates was detected by a pThr antibody [44].

### Immunoblot analysis

To exam the stability of cMyc-ANAC078 protein, pEGAD-4XcMyc-ANAC078 was transformed into WT and *pi4kγ5–1*, respectively. Due to the low protein level of ANAC078 protein in the transgenic lines (overexpression of ANAC078 leads to dwarf plants), 30-day-old plants were pretreated with 50 μM MG132 (Sigma, USA) for 3 h. Protein extracted from transgenic plants were re-suspended in the extraction buffer (20 mM Tris-HCl, pH 7.5, 150 mM NaCl, 0.5% Tween-20, 1 mM EDTA, 1 mM DTT) containing a protease inhibitor cocktail (Roche, Germany). After addition of an equal volume of 2X SDS buffer, the samples were boiled for 5 min, fractionated by 10% SDS-PAGE and transferred to a PVDF membrane by semi-dry blotting. The blots were incubated with a mouse anti-cMyc antibody (Millipore, USA) and then with a bovine anti-mouse IgG AP-conjugated secondary antibody (Santa Cruz Biotechnology, USA). AP activity was detected by the BCIP/NBT Detection Reagents (Invitrogen, USA).

### Yeast two-hybrid analysis

Coding sequence of *PI4Kγ5* was amplified (primers PI4Kγ5–11 and PI4Kγ5–12) and subcloned into pGBKT7 or pGADT7 vectors. The pGBKT7-ANAC078 construct was generated using primers ANAC078-9/ANAC078-10. For yeast two-hybrid screening, BD-PI4Kγ5 was used as bait to screen the candidate interacting proteins on SD (-Leu/-Trp/-His/-Ade, Clontech, USA) plates.

For auxotroph assays, candidate clones were streaked onto SD (-Leu/-Trp/-His) medium supplemented with X-α-Gal (80 mg/L) and grown at 30°C for 4 days.

### Spilt-Luciferase assays

Coding regions of *PI4Kγ5* and *ANAC078* were fused with nLuc or cLuc, respectively. Constructs PI4Kγ5-nLuc (primers PI4Kγ5-13/PI4Kγ5–14), ANAC078-cLuc (primers ANAC078-11/ANAC078-12), SGT1-cLuc, and RAR1-nLuc were respectively transformed into *Agrobacterium*. The positive clones were then mixed and injected into *Nicotiana benthamiana* leaves and observed after 48 h according to previous description [45].

### Co-localization studies of PI4Kγ5 and ANAC078

Coding regions of *ANAC078* (primers ANAC078-5 and ANAC078-6) and *PI4Kγ5* (primers PI4Kγ5–7 and PI4Kγ5–8) were amplified and subcloned into vector pA7 (N-terminus fusion with GFP) or pRTL2 respectively, resulting in the GFP-ANAC078 and PI4Kγ5-RFP fusion constructs, which were transiently expressed in *Arabidopsis* protoplasts by PEG/CaCl_2_ methods [46]. The fluorescence was observed by confocal laser scanning microscopy (Olympus FV1000).

### Electrophoretic Mobility Shift Assay (EMSA)

DNA fragments of *YUC2*, *YUC4* and *GH3*.*5* promoters were PCR-amplified (primers YUC2-3/YUC2-4, YUC4-3/YUC4-4, GH3.5-3/GH3.5–4) and resultant fragments were labeled with DIG probe synthesis kit (Roche, Germany). Unlabeled DNAs were used as the cold competitor. Labeled DNAs were incubated without or with unlabeled DNA (2–16 fold) and recombinant ANAC078-His fusion proteins (20–100 ng) in binding buffer (75 mM HEPES, 175 mM KCl, 5 mM EDTA, 40% glycerol, 5 mM DTT, 1 mM MgCl_2_) at room temperature for 20 min, then run on a 0.5X 5% polyacrylamide gel and followed by membrane transfer, cross-linking, blocking, and antibody incubation. The binding was detected with disodium 3-(4-methoxyspiro{1,2-dioxetane-3,2′-(5’-chloro)tricycle [3.3.1.13,7]decan}-4-yl)phenyl phosphate (CSPD, Roche, Germany) according to the manufacturer’s manual [47].

### Chromatin immunoprecipitation (ChIP) assay

Transgenic WT seedlings overexpressing YFP-ANAC078 (primers ANAC078-15, ANAC078-16, ANAC078-17, ANAC078-20) fusion protein were used for ChIP assay using EpiQuik^™^ Plant ChIP Kit (Epigntek, USA). Primers (YUC2-5/YUC2-6, YUC4-5/YUC4-6, GH3.5-5/GH3.5–6) specific for *YUC2*, *YUC4*, and *GH3*.*5* promoter region were used for PCR amplification using ChIP populations from immunoprecipitations or control as templates.

### Dual-luciferase transient expression assay using *Arabidopsis* protoplasts

Isolation and transformation of *Arabidopsis* mesophyll protoplasts were previously described [46]. Promoter of *YUC2* (-2965 bp upstream to -32 bp of initiation of translation ATG) was amplified using genomic DNA as template (with primers YUC2-7 and YUC2-8) and subcloned into a modified pGreen0800 vector [48]. Resultant construct and construct expressing GFP-ANAC078 fusion protein were transformed into *Arabidopsis* protoplasts by PEG/CaCl_2_ methods. Transformed protoplasts were collected for the dual-luciferase assay using Dual-Luciferase Reporter Assay System (Promega, USA).

## Supporting Information

S1 Fig Identification of the *pi4kγ5–1* mutant, which presents similar area and cell size of leaves compared to WT. A. The T-DNA fragment is inserted 35-bp upstream of 5’ UTR of *PI4Kγ5*. B. Similar area of the 7^th^ and 8^th^ rosette leaves of WT and *pi4kγ5–1*. Bar = 1 cm. C. Similar cell size of WT and *pi4kγ5–1* leaves. The palisade cell size was observed by interference microscope. Bars = 50 μm.
(TIF)

S2 Fig Enhanced auxin accumulation at tip of the 7^th^ rosette leaf along with leaf development. Leaves at different stages (indicated by different sizes) of *DR5-GUS* or *pi4kγ5–1 DR5-GUS* lines were observed.
(TIF)

S3 Fig L-Kyn treatment resulted in the significantly reduced GUS activity of *pi4kγ5–1 DR5-GUS* lines. Seedlings were grown on MS medium supplemented with Kyn (1 μM, DMSO was used as control) for 30 days and the 7^th^ rosette leaf was stained and observed. Arrows highlighted the GUS activity (accumulation of auxin) at leaf tip and serration. The experiments were repeated three times. Bars = 1 mm.
(TIF)

S4 Fig Yeast two-hybrid analysis identified ANAC078 an interacting protein of PI4Kγ5. A. BD-PI4Kγ5 was used for yeast two-hybrid screening and interaction of two candidate clones harboring ANAC078 cDNA (1, 2; clones 4 and 5 are two other proteins and used as negative control) with PI4Kγ5 was confirmed by observing the cell growth on synthetic dropout (SD) medium lacking Leu, Trp, His, and Ade (SD-Trp-Leu-His-Ade) after co-transformation. B. A schematic diagram of ANAC078 protein, ANAC078 protein deletion of transmembrane (TM) region [ANAC078(-TM)] and ANAC078 protein deletion of C-terminus [ANAC078(-C)].
(TIF)

S5 Fig Increased transcription levels of genes related to flavonoid biosynthesis of *pi4kγ5–1* under high light. Expressions of genes related to flavonoid biosynthesis under high light were examined using WT, *anac078* and *pi4kγ5–1* plants. Two-week-old *Arabidopsis* plants grown under normal condition (100 μmol m^−2^ s^−1^) were exposed to high light (HL, 600 μmol m^−2^ s^−1^, 30°C) for 1 h, then collected for qPCR analysis. The experiments were repeated three times and data are presented as means ± SE (n = 3).
(TIF)

S6 Fig *ANAC078* is expressed highly in stems and leaves and *pi4kγ5–1 anac078* double mutant present phenotype similar to *pi4kγ5–1*. A. qPCR analysis revealed that *ANAC078* is expressed in various tissues, and relatively highly expressed in stems and leaves. The *ACTIN7* gene was used as an internal reference and transcription level of *ANAC078* in seedlings was set as 1.0. The experiments were repeated three times and data are presented as means ± SE (n = 3). B. Identification of homozygous *pi4kγ5–1 anac078* double mutant. Primers PI4Kγ5-1/PI4Kγ5–2, ANAC078-1/ANAC078-2, that locate each side of the T-DNA were used for PCR analysis. C. Semi-quantitative RT-PCR analysis confirmed the deficiency of *ANAC078* transcription in *pi4kγ5–1 anac078* homozygous double mutant. D. Homozygous *pi4kγ5–1 anac078* double mutant showed highly serrated rosette leaves and abnormal petals similar as *pi4kγ5–1*. Bar = 2 mm. E. *anac078* mutant present indistinguishable growth compared to WT. Seedling overexpressing ANAC078 (cMyc-ANAC078 in WT) show smaller rosette leaves, but no serrated margins in adult leaves. The 7th leaf was observed and shown. Bar = 1 cm.
(TIF)

S1 Table Statistics of the segregation ratio of back-cross F2 plants exhibiting serrated leaf or normal phenotype. (DOC)

S2 Table Primers used for mutant genotyping and plasmid construction. (DOC)
