# Supplementary figures and images for: Arabidopsis Type II Phosphatidylinositol 4-Kinase PI4Kγ5 Regulates Auxin Biosynthesis and Leaf Margin Development through Interacting with Membrane-Bound Transcription Factor ANAC078
Source: PLoS Genet. 2016 Aug 16;12(8):e1006252. doi: 10.1371/journal.pgen.1006252 (PMC4986951; doi:10.1371/journal.pgen.1006252)

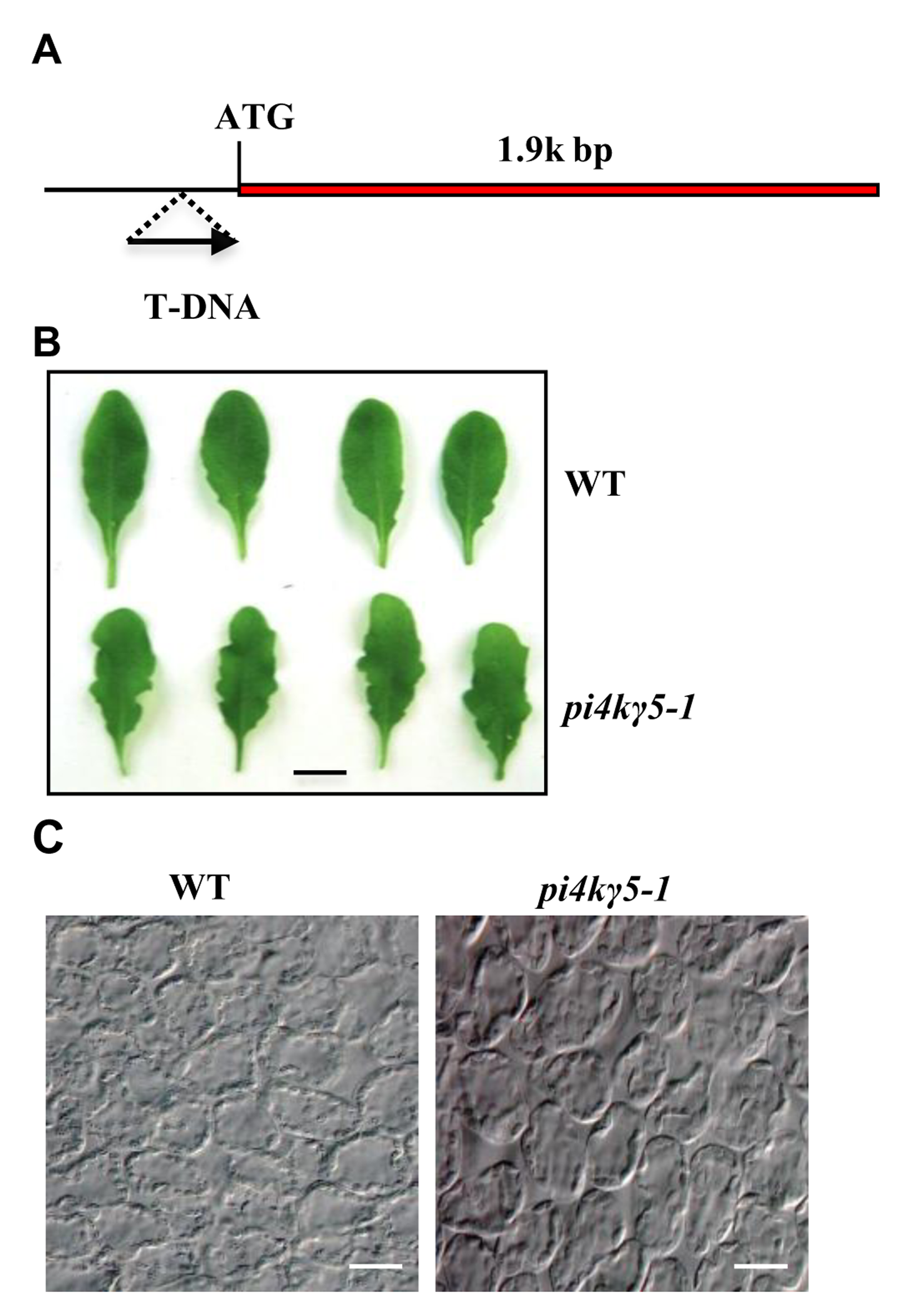

Supplement: S1 Fig — A. The T-DNA fragment is inserted 35-bp upstream of 5’ UTR of PI4Kγ5. B. Similar area of the 7th and 8th rosette leaves of WT and pi4kγ5–1. Bar = 1 cm. C. Similar cell size of WT and pi4kγ5–1 leaves. The palisade cell size was observed by interference microscope. Bars = 50 μm. (TIF) [file pgen.1006252.s001.tif]

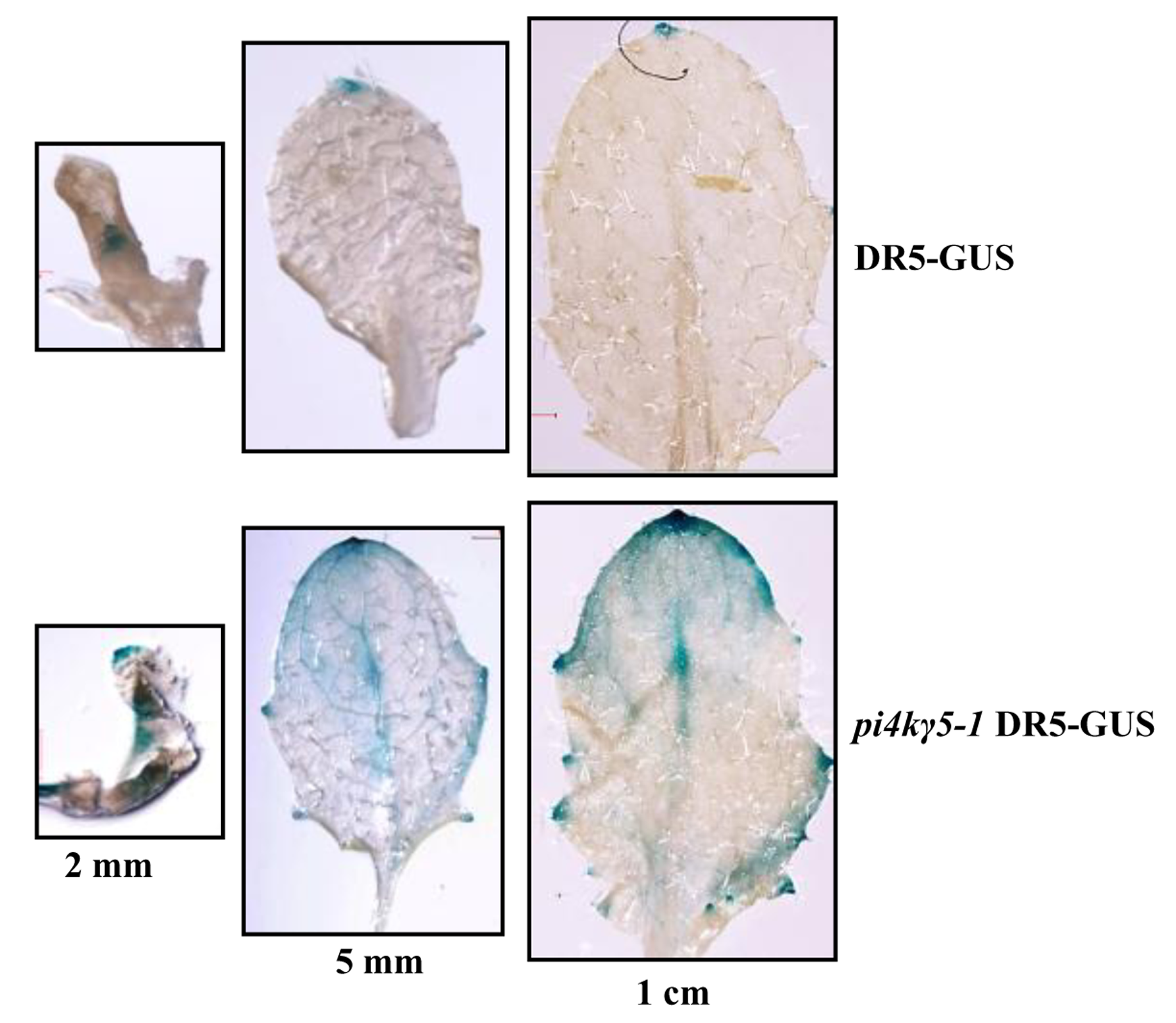

Supplement: S2 Fig — Leaves at different stages (indicated by different sizes) of DR5-GUS or pi4kγ5–1 DR5-GUS lines were observed. (TIF) [file pgen.1006252.s002.tif]

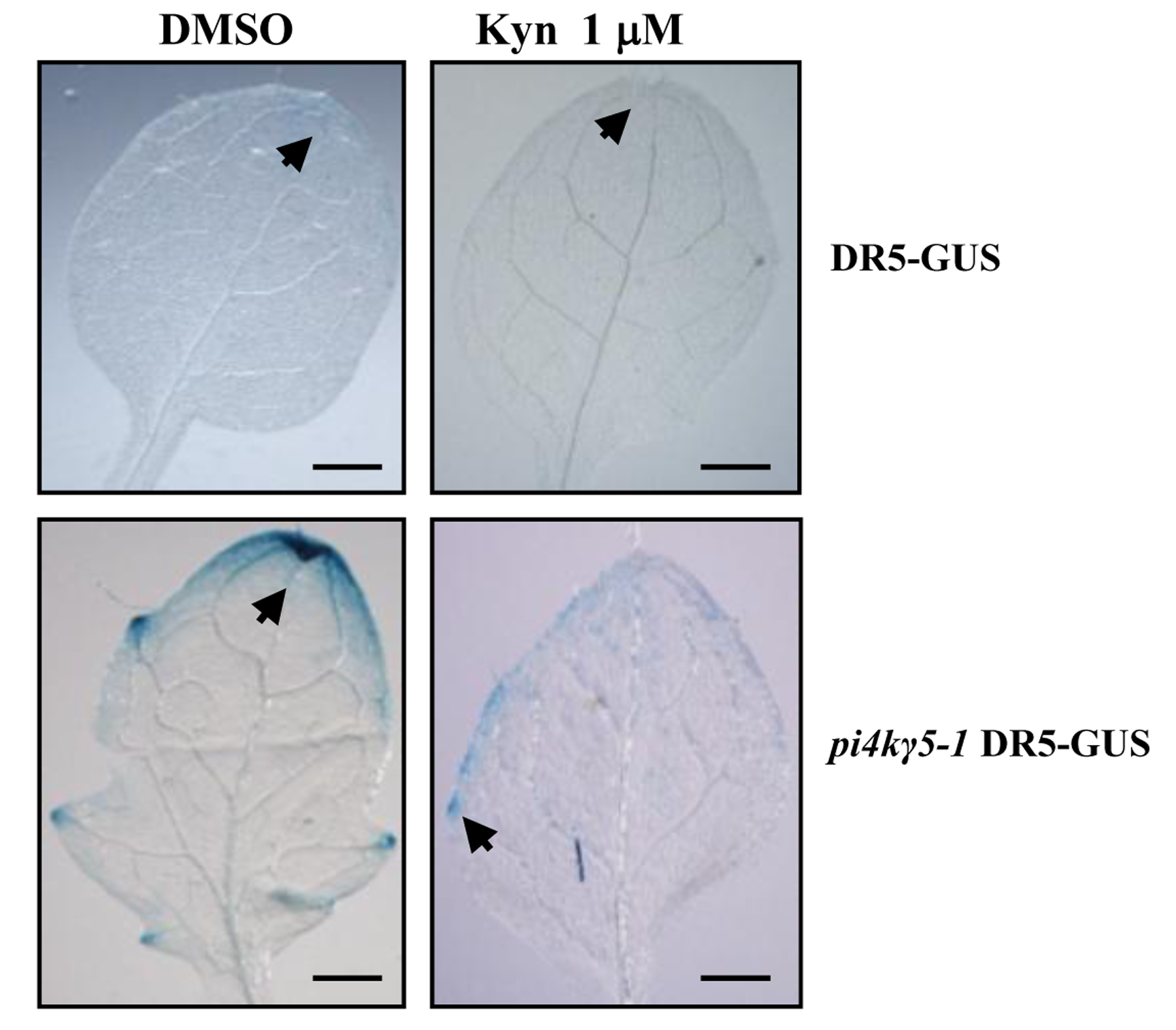

Supplement: S3 Fig — Seedlings were grown on MS medium supplemented with Kyn (1 μM, DMSO was used as control) for 30 days and the 7th rosette leaf was stained and observed. Arrows highlighted the GUS activity (accumulation of auxin) at leaf tip and serration. The experiments were repeated three times. Bars = 1 mm. (TIF) [file pgen.1006252.s003.tif]

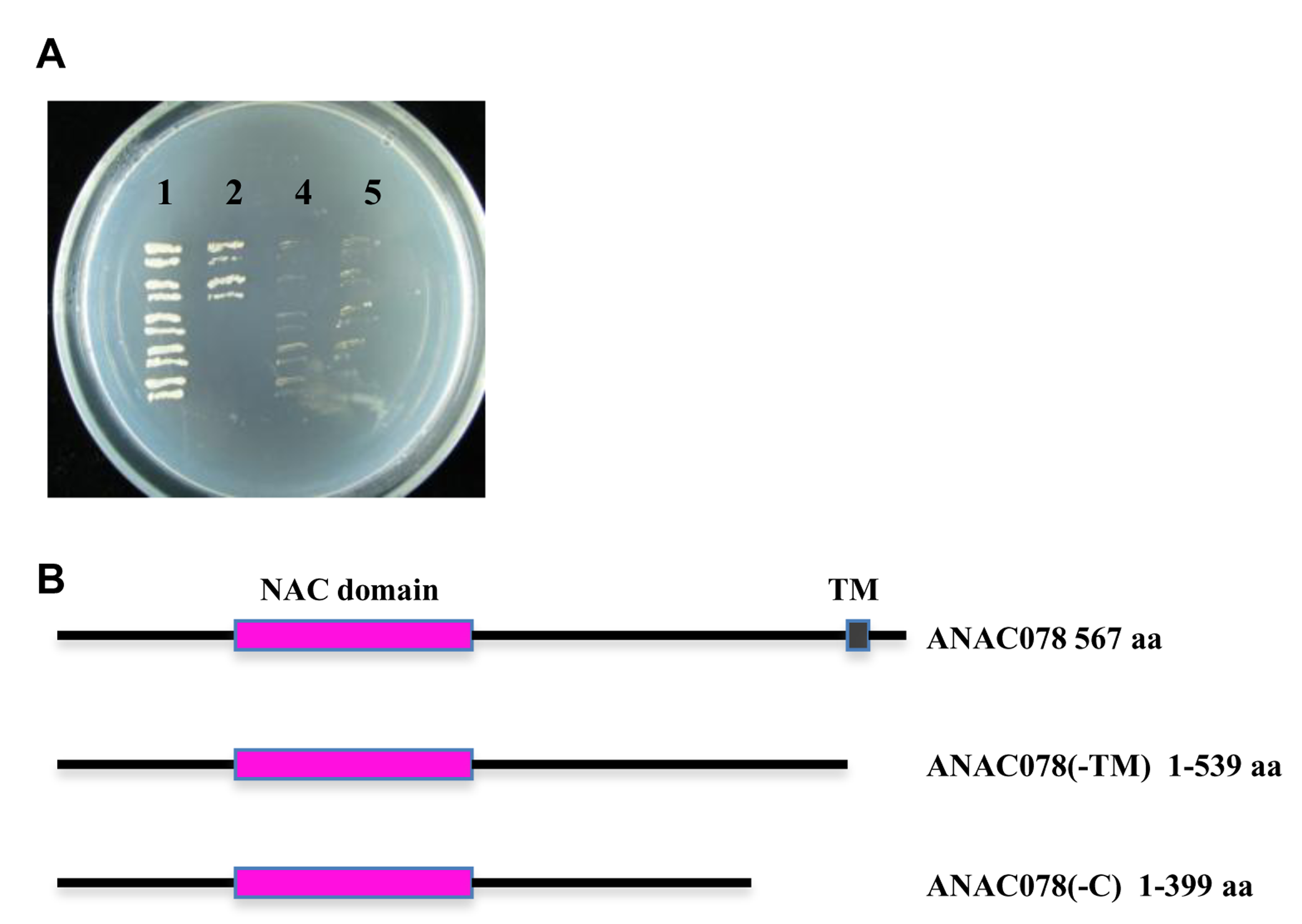

Supplement: S4 Fig — A. BD-PI4Kγ5 was used for yeast two-hybrid screening and interaction of two candidate clones harboring ANAC078 cDNA (1, 2; clones 4 and 5 are two other proteins and used as negative control) with PI4Kγ5 was confirmed by observing the cell growth on synthetic dropout (SD) medium lacking Leu, Trp, His, and Ade (SD-Trp-Leu-His-Ade) after co-transformation. B. A schematic diagram of ANAC078 protein, ANAC078 protein deletion of transmembrane (TM) region [ANAC078(-TM)] and ANAC078 protein deletion of C-terminus [ANAC078(-C)]. (TIF) [file pgen.1006252.s004.tif]

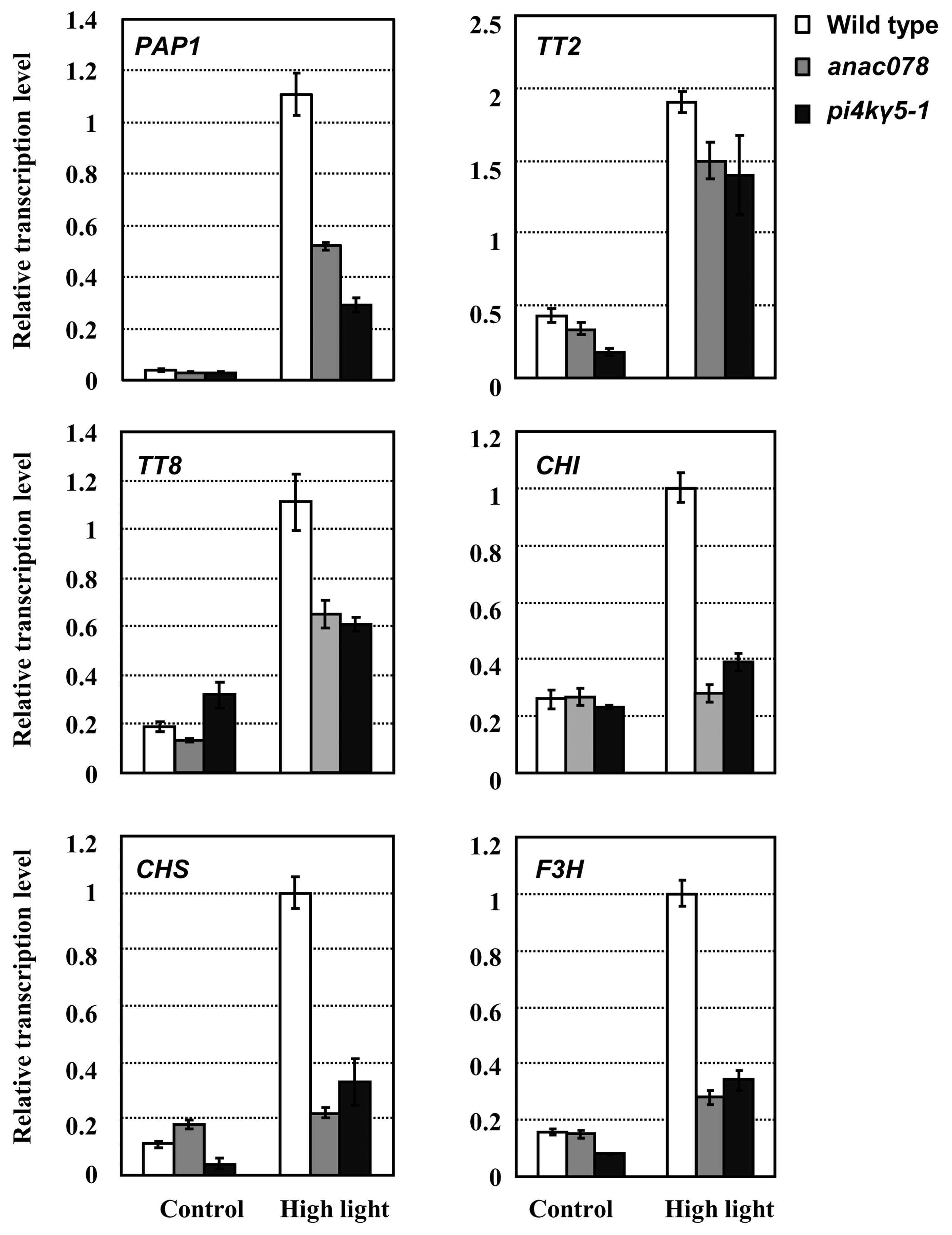

Supplement: S5 Fig — Expressions of genes related to flavonoid biosynthesis under high light were examined using WT, anac078 and pi4kγ5–1 plants. Two-week-old Arabidopsis plants grown under normal condition (100 μmol m−2 s−1) were exposed to high light (HL, 600 μmol m−2 s−1, 30°C) for 1 h, then collected for qPCR analysis. The experiments were repeated three times and data are presented as means ± SE (n = 3). (TIF) [file pgen.1006252.s005.tif]

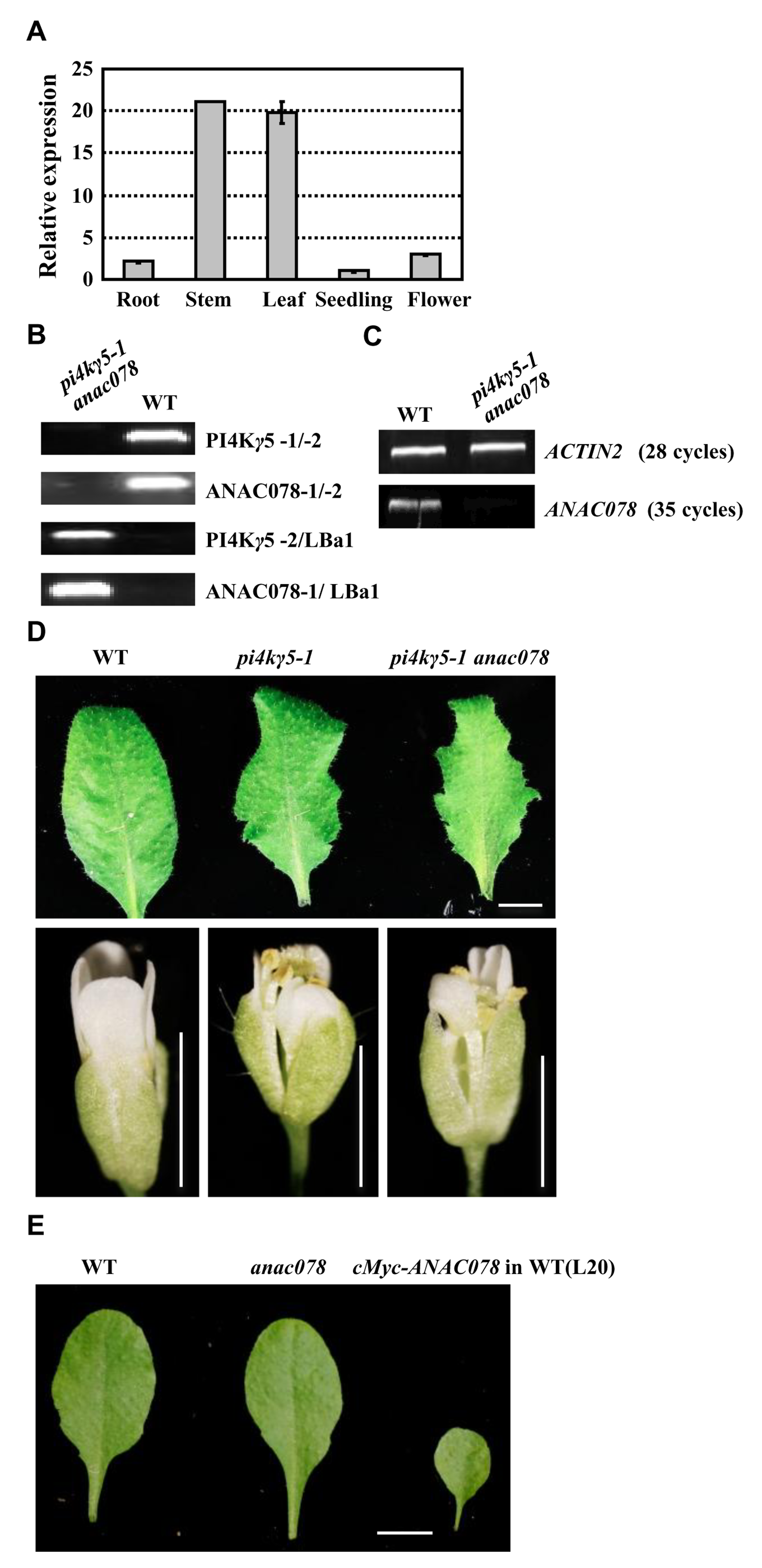

Supplement: S6 Fig — A. qPCR analysis revealed that ANAC078 is expressed in various tissues, and relatively highly expressed in stems and leaves. The ACTIN7 gene was used as an internal reference and transcription level of ANAC078 in seedlings was set as 1.0. The experiments were repeated three times and data are presented as means ± SE (n = 3). B. Identification of homozygous pi4kγ5–1 anac078 double mutant. Primers PI4Kγ5-1/PI4Kγ5–2, ANAC078-1/ANAC078-2, that locate each side of the T-DNA were used for PCR analysis. C. Semi-quantitative RT-PCR analysis confirmed the deficiency of ANAC078 transcription in pi4kγ5–1 anac078 homozygous double mutant. D. Homozygous pi4kγ5–1 anac078 double mutant showed highly serrated rosette leaves and abnormal petals similar as pi4kγ5–1. Bar = 2 mm. E. anac078 mutant present indistinguishable growth compared to WT. Seedling overexpressing ANAC078 (cMyc-ANAC078 in WT) show smaller rosette leaves, but no serrated margins in adult leaves. The 7th leaf was observed and shown. Bar = 1 cm. (TIF) [file pgen.1006252.s006.tif]
