# Supplementary material for: Arabidopsis Type II Phosphatidylinositol 4-Kinase PI4Kγ5 Regulates Auxin Biosynthesis and Leaf Margin Development through Interacting with Membrane-Bound Transcription Factor ANAC078
Source: PLoS Genet. 2016 Aug 16;12(8):e1006252. doi: 10.1371/journal.pgen.1006252 (PMC4986951; doi:10.1371/journal.pgen.1006252)
Supplement: S1 Table — (DOC) [file pgen.1006252.s007.doc]

**S1 Table. Statistics of the segregation ratio of back-cross F2 plants exhibiting serrated leaf or normal phenotype.** Seeds of independent F1 heterozygous plants (lines 2, 5 and 17, confirmed by PCR analysis) from back cross (*pi4kγ5-1* X WT) were sterilized and germinated on MS plates. Phenotype of F2 adult plants were recorded and analyzed.

|  |  | Wild type | Homozygous lines |
| --- | --- | --- | --- |
| Line 2 | 75 | 22 (1/3.4) | 17 (1/4.4) |
| Line 5 | 91 | 15 (1/6) | 19 (1/4.8) |
| Line 17 | 93 | 26 (1/3.6) | 18 (1/5.2) |
| Total | 259 | 63 (1/4.11) | 54 (1/4.79) |
