# Supplementary material for: Arabidopsis Type II Phosphatidylinositol 4-Kinase PI4Kγ5 Regulates Auxin Biosynthesis and Leaf Margin Development through Interacting with Membrane-Bound Transcription Factor ANAC078
Source: PLoS Genet. 2016 Aug 16;12(8):e1006252. doi: 10.1371/journal.pgen.1006252 (PMC4986951; doi:10.1371/journal.pgen.1006252)
Supplement: S2 Table — (DOC) [file pgen.1006252.s008.doc]

**S2 Table. Primers used for mutant genotyping and plasmid construction.** Added restriction endonuclease sites are underlined.

| **Primers** | **Sequences (5'-3')** | **Restriction enzymes** |
| --- | --- | --- |
| PI4Kγ5-1 | CCTCGGTTGTTTGTTTCC |  |
| PI4Kγ5-2 | GACCCACAGGTGTCTTCATT |  |
| PI4Kγ5-3 | GCCGAGTGAGATTGAAGTGG |  |
| PI4Kγ5-4 | GTGAGTCCGAGTGGTAATGC |  |
| PI4Kγ5-5 | CCCAAGCTTGGGTCAGCACCTCTACTACAT | *Hind*III |
| PI4Kγ5-6 | CGGGATCCGAGTGTGGAGACAAAAAA | *BamH*I |
| PI4Kγ5-7 | GGGGTACCATGTCACGTAAGCTTGACAG | *Kpn*I |
| PI4Kγ5-8 | GAAGATCTTCA AAACTGACAAGAAGT | *BglII* |
| PI4Kγ5-9 | ACGCGTCGACAAATGTCACGTAAGCTTGACAG | *Sal*I |
| PI4Kγ5-10 | CCGCTCGAG AAACTGACAAGAAGTCCCCA | *Xho*I |
| PI4Kγ5-11 | GGAATTCATGTCACGTAAGCTTGACAG | *EcoR*I |
| PI4Kγ5-12 | CGGGATCCACTGGATCAATGCCCTTCT | *BamH*I |
| PI4Kγ5-13 | ACGCGTCGACAAACTGACAAGAAGTCCCCA | *Sal*I |
| PI4Kγ5-14 | GGGGTACCATGTCACGTAAGCTTGACAG | *Kpn*I |
| ANAC078-1 | CGTCTACACGCAGCACAA |  |
| ANAC078-2 | ACTGGAGTCAGGGCAATC |  |
| ANAC078-3 | GAGGTCGCAGGAGGCAAAGA |  |
| ANAC078-4 | CGTCCTTTGTTTGGAACTGTGA |  |
| ANAC078-5 | GCTCTAGACATGGGTCGTGGCTCAGT | *Xba*I |
| ANAC078-6 | CGGGATCCTTACCGAGCAGACACCAT | *BamH*I |
| ANAC078-7 | GGAATTCATGGGTCGTGGCTCAGT | *EcoR*I |
| ANAC078-8 | CCGCTCGAGCCGAGCAGACACCATGGT | *Xho*I |
| ANAC078-9 | GGAATTCCATATGATGGGTCGTGGCTCAGT | *Nde*I |
| ANAC078-10 | GGAATTCTCTTAGGACCCGTACCAC | *EcoR*I |
| ANAC078-11 | GGGGTACCATGGGTCGTGGCTCAGTG |  |
| ANAC078-12 | ACGCGTCGACTTACCGAGCAGACACCAT |  |
| ANAC078-13 | CCGCTCGAGCGCCGTAATTCCTGTCTT | *Xho*I |
| ANAC078-14 | CCGCTCGAGCTGGTCAAGGGCTTCTTG | *Xho*I |
| ANAC078-15 | GGGGTACCATGGTGAGCAAGGGCGAG | *Kpn*I |
| ANAC078-16 | GACTAGTCTTGTACAGCTCGTCCAT | *Spe*I |
| ANAC078-17 | GACTAGTATGGGTCGTGGCTCAGT | *Spe*I |
| ANAC078-18 | GCTCTAGATTACCGAGCAGACACCAT | *Xba*I |
| ANAC078-19 | CCCAAGCTTATGGGTCGTGGCTCAGT | *Hind*III |
| ANAC078-20 | CCGCTCGAGCTGGTCAAGGGCTTCTTG | *Xho*I |
| YUC2-1 | GAAAGGCGAAAGCGGATTGT |  |
| YUC2-2 | GGACGAGCCAATGGTAAGAA |  |
| YUC2-3 | TTTCCAAAATAGCATGTTCCA |  |
| YUC2-4 | GCTAATCAGTAAAATGATTACATGA |  |
| YUC2-5 | GGTAGCTAGCTCAGTGGT |  |
| YUC2-6 | TGTGATATTCTCGGTATG |  |
| YUC2-7 | ACGCGTCGACGCATAATCAAATTTTAGTTAC | *SalI* |
| YUC2-8 | CGGGATCCACAATGTTGAGGACGAGCCAATGG | *BamHI* |
| YUC4-1 | GGGCAAAGTTTCTGAATGGG |  |
| YUC4-2 | GTCCTCTTCTCGTGAAACCC |  |
| YUC4-3 | CTCTCTCACTCACTCTCGAGCA |  |
| YUC4-4 | TTGGAAATTATATAGAGACCACGTA |  |
| YUC4-5 | CTCTCTCACTCACTCTCG |  |
| YUC4-6 | TGCACCTAAGAAGTGTCC |  |
| GH3.5-3 | CTAGTAGAAATAAAATAA |  |
| GH3.5-4 | GACCAGACAGCTGGACTC |  |
| GH3.5-5 | TGCCGGTGATTTGTGGGA |  |
| GH3.5-6 | TAACTATCTCAATCAGAC |  |
| LBa1 | TGGTTCACGTAGTGGGCCATCG |  |
| H4-1 | ATGTCAGGTCGTGGAAAGGG |  |
| H4-2 | TCCGAGCGTGCTCAGTGTAA |  |
| H4-3 | TCTCTATGGATTCGGTGGTTGA |  |
| H4-4 | CCGGAACAACATTACAACGCAAAC |  |
| PAP1-1 | CTGCGAAAAGGTGCTTGGA |  |
| PAP1-2 | CCCAGCTCTTACAGGAACTTGG |  |
| TT2-1 | CTTGGAAACAGATGGTCGTTGATAG |  |
| TT2-2 | GAGTTCCAATGATTCTTTATTTCATTGTC |  |
| TT8-1 | CAACAGCTCAGGGAGCTTTATGA |  |
| TT8-2 | GCGGTGCATGCTCTTGCT |  |
| CHI-1 | GGATCGCTGTCATCGAGAACA |  |
| CHI-2 | CACCGTTCTTCCCGATGATAGA |  |
| CHS-1 | CCTGCAGGCATCTTGGCTATT |  |
| CHS-2 | CACTGTTGGTGATGCGGAAGT |  |
| F3H-1 | GAAGGAGCGTTTGTCGTCAATC |  |
| F3H-2 | CAGCATTCTTGAACCTCCCATT |  |
| ACTIN7-1 | CCGGTATTGTGCTCGATTCTG |  |
| ACTIN7-2 | TTCCCGTTCTGCGGTAGTGG |  |
